# Dynamic anoxic ferruginous conditions during the end-Permian mass extinction and recovery

**DOI:** 10.1038/ncomms12236

**Published:** 2016-07-19

**Authors:** M. O. Clarkson, R. A. Wood, S. W. Poulton, S. Richoz, R. J. Newton, S. A. Kasemann, F. Bowyer, L. Krystyn

**Affiliations:** 1 School of GeoSciences, University of Edinburgh, James Hutton Road, Edinburgh EH9 3FE, UK; 2 School of Earth and Environment, University of Leeds, Leeds LS2 9JT, UK; 3 Institute of Earth Sciences, NAWI Graz, University of Graz, Heinrichstraße 26, 8010 Graz, Austria; 4 Department of Geosciences and MARUM-Center for Marine Environmental Sciences, University of Bremen, 28334 Bremen, Germany; 5 Institute for Palaeontology, Vienna University, Althanstrasse 14, 1090 Vienna, Austria

## Abstract

The end-Permian mass extinction, ∼252 million years ago, is notable for a complex recovery period of ∼5 Myr. Widespread euxinic (anoxic and sulfidic) oceanic conditions have been proposed as both extinction mechanism and explanation for the protracted recovery period, yet the vertical distribution of anoxia in the water column and its temporal dynamics through this time period are poorly constrained. Here we utilize Fe–S–C systematics integrated with palaeontological observations to reconstruct a complete ocean redox history for the Late Permian to Early Triassic, using multiple sections across a shelf-to-basin transect on the Arabian Margin (Neo-Tethyan Ocean). In contrast to elsewhere, we show that anoxic non-sulfidic (ferruginous), rather than euxinic, conditions were prevalent in the Neo-Tethys. The Arabian Margin record demonstrates the repeated expansion of ferruginous conditions with the distal slope being the focus of anoxia at these times, as well as short-lived episodes of oxia that supported diverse biota.

## Abstract (web summary)

Oceanic anoxia is invoked for driving the Permo-Triassic Mass Extinction, but the timing, distribution and chemical state are poorly understood. Here, the authors show that fluctuations of anoxic, non-sulfidic (ferruginous) conditions were important for the delayed biotic recovery in the Neo-Tethys.

## Graphical abstract

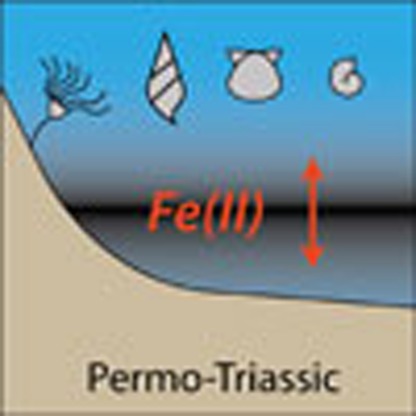

Phanerozoic oceanic anoxic events (OAEs) represent extreme periods of Earth history that record major disturbances to the carbon cycle^1^. OAEs typify greenhouse climate states and have played a major role in dictating evolution and extinction events^1^. In particular, euxinic (anoxic and sulfidic) water column conditions have been implicated as a major driver for the end-Permian mass extinction^2^,^3^,^4^,^5^,^6^, which represents the greatest mass extinction of the Phanerozoic with a uniquely protracted and complex ∼5 Myr recovery period^7^. Once described as a ‘superanoxic' event that lasted for ∼20 Myr (ref. 8), our understanding of the Permian Triassic interval has since been refined by evidence for changes in the spatial and temporal distribution, and intensity, of anoxia^9^,^10^,^11^. These studies highlight recurrent anoxia that has an unresolved and complex relationship to both evolutionary dynamics and the global carbon cycle.

Refined techniques for identifying the precise nature of ocean redox conditions^12^ have increasingly highlighted the prevalence of anoxic non-sulfidic (ferruginous conditions) through Earth history^13^, including during Phanerozoic periods of anoxia^14^,^15^. However, despite suggestions of the extreme redox state of euxinia^4^,^6^, no redox studies have yet employed methods capable of distinguishing anoxic ferruginous conditions from euxinic conditions across the full Permo-Triassic extinction and recovery interval. This is critical as the precise nature of water column chemistry during periods of anoxia has profound implications for the evolution of the biosphere and for feedbacks associated with the biogeochemical cycling of elements such as nitrogen^16^ and phosphorus^14^ in the sediment and water column. In addition, no study has yet applied redox proxies of any kind to produce a vertically resolved record of the entire water column, even though this is crucial for understanding anoxic events^17^,^18^. Without such records, a detailed causal understanding of the relationship between anoxia, euxinia, extinction events and wider carbon cycle processes is not possible.

To address this, we have undertaken Fe-speciation, Fe/Al and pyrite S-isotope (δ^34^S_pyrite_) analyses of a suite of sediments covering a shelf to basin depth transect across the Arabian Margin (Fig. 1), in the central Neo-Tethyan Ocean Our sites include shelf carbonates^19^,^20^,^21^, continental slope sediments (Sumeini Group^22^,^23^), basinal deposits (Hawasina Basin^24^) and offshore highs (Ba'id^25^,^26^). Detailed section descriptions are given in Supplementary Note 1. Our samples primarily span from the Late Permian (∼260 Ma), across the extinction interval (EI) and the Permian Triassic Boundary (PTB), to the Late Spathian. This unique depth transect allows for a particularly well-resolved vertical and temporal record of regional redox dynamics within an established carbon isotope and biostratigraphic framework^20^,^23^,^24^,^27^.We identify a temporally and spatially dynamic redox system that shows expansions of anoxic ferruginous conditions across the Arabian Margin. Expansions occur at the EI, the Dienerian/Smithian boundary and during the Smithian/Spathian boundary, with the distal slope, rather than the deep basin, being the focus of anoxia at these times. Our data also demonstrate short-lived interludes of oxic water column conditions, which in contrast to ferruginous episodes are characterized by a diverse fossil record, suggesting that biotic recovery in the Neo-Tethys was rapid during these oxygenation episodes.

## Results

### Fe systematics

Fe-speciation is a widely utilized proxy for regional water column redox conditions^12^,^18^, being able to differentiate oxic from anoxic ferruginous and euxinic conditions. This sequential chemical extraction technique gives a measure of highly reactive Fe to total Fe (Fe_HR_/Fe_T_). Fe_HR_ refers to Fe minerals that are considered highly reactive towards biological and abiological reduction under anoxic conditions, and includes carbonate-associated Fe (Fe_carb_; for example, ankerite and siderite), ferric (oxyhydr)oxides (Fe_ox_; for example, goethite and haematite), magnetite Fe (Fe_mag_) and Fe sulfide minerals (Fe_py_; for example, makinawite and pyrite).

Fe_HR_/Fe_T_ ratios of <0.22 provide robust evidence for deposition from an oxic water column^13^,^28^. Anoxic water column conditions are identified when Fe_HR_/Fe_T_ ratios are >0.38, which result from the additional water column formation of either Fe sulfides under euxinic conditions, or non-sulfidized Fe minerals under ferruginous conditions^29^,^30^. Fe_Py_/Fe_HR_ ratios can then distinguish ferruginous (<0.7–0.8) from euxinic (>0.7–0.8) conditions^13^,^28^,^31^,^32^. Values of Fe_HR_/Fe_T_ between 0.22 and 0.38 are somewhat equivocal, and may represent either oxic or anoxic deposition^28^,^33^,^34^. In the latter case, diagenetic transfer of Fe_HR_ minerals to Fe-rich clay minerals, or rapid sedimentation, can diminish Fe_HR_ enrichments. During diagenetic transformation of Fe_HR_ to unreactive Fe minerals (Fe_UR_), the total Fe enrichment is still preserved and hence we also utilize Fe/Al ratios to provide further information on water column redox conditions^18^,^33^, where oxic marine sediments have average ratios of 0.55±0.11 (refs 34, 35). Dilution of Fe_HR_ by rapid sedimentation does not appear to have affected our samples (Supplementary Note 2). Oxidative weathering of samples could potentially mask Fe_py_ enrichments but would not alter Fe/Al ratios. The high proportion of Fe_carb_ in our samples, however, argues against this process significantly affecting our results (Supplementary Note 3).

**Figure 2:**
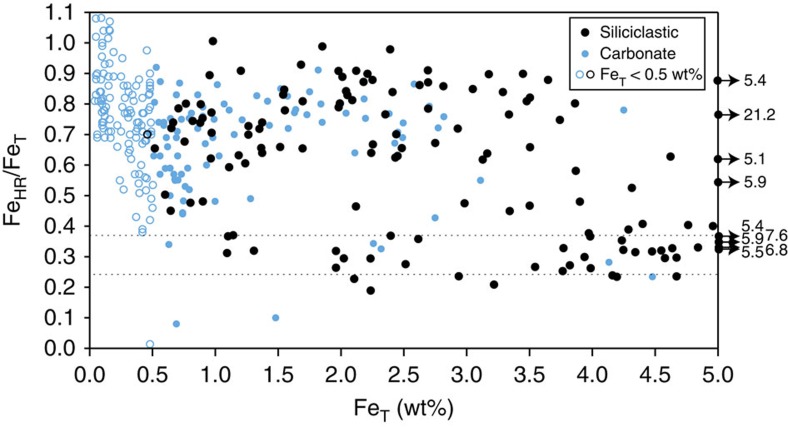
Distribution of Fe-speciation data as a function of total Fe. Samples are coloured by lithology (black=siliciclastic, blue=carbonate). Dashed lines are at 0.22 and 0.38, marking the interpretive thresholds for Fe-speciation. Carbonate samples with Fe_T_<0.5 wt%. (open blue circles) have Fe_HR_/Fe_T_ ratios almost exclusively>0.38, suggesting spurious enrichments of Fe_HR_ similar to modern sediments^35^. Fe_T_ and Fe-speciation data for these samples cannot be interpreted. As Fe_T_ decreases there may also be a decrease in precision, leading to excessively high Fe_HR_/Fe_T_ values>1. Extreme values have therefore been removed from the data set.

**Figure 3:**
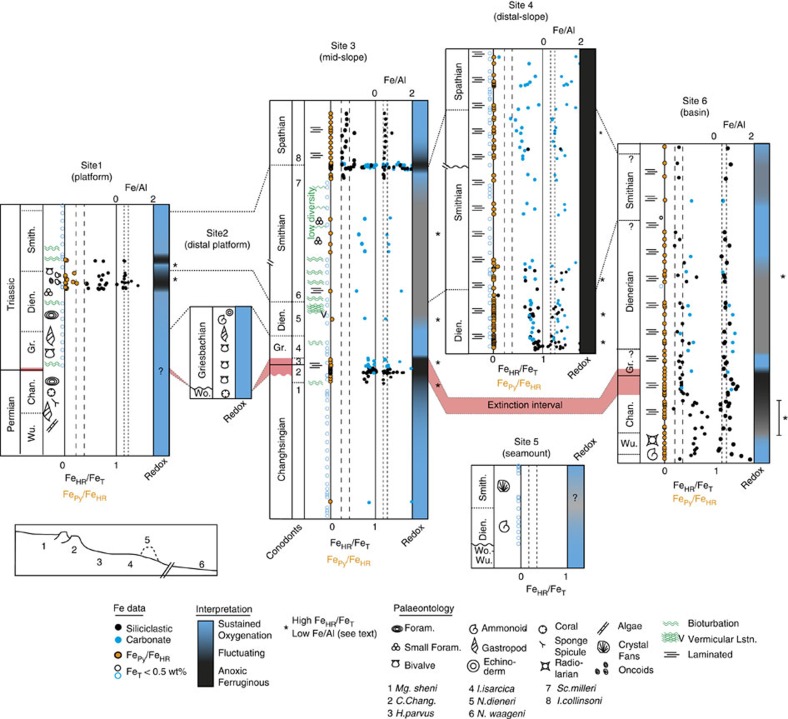
Summary of Fe-speciation and Fe/Al records for the Arabian Margin. Samples where Fe_T_>0.5 wt% (closed circles) can be used confidently for Fe-speciation regardless of lithology^35^. Samples with Fe_T_<0.5 wt% (open circles) cannot be interpreted for Fe_HR_/Fe_T_ ratios as they may show spurious results^35^ (see text), but we nevertheless show the points at which these samples were taken. Data point colour identifies lithology for both Fe_T_ categories. Palaeontological information^27^,^36^ is summarized and is particularly useful for identifying oxygenated conditions when Fe_T_<0.5 wt%. Fe/Al is used to help interpret Fe_HR_/Fe_T_ values between 0.22 and 0.38 (see text for discussion). Fe/Al scale is maximized at 2, values >2 can be seen in Supplementary Figs 1–7. Lines are shown at 0.22 and 0.38 for Fe_HR_/Fe_T_ ratios, 0.44 and 0.66 (0.55±0.11) for Fe/Al. Sections are scaled for clarity with section breaks indicated corresponding to Supplementary Figs 1–7. Fe-speciation was not undertaken for site 2. Extinction interval shown in red. Not all conodont zones, as defined in South China, are identifiable in the Central Tethys, but key conodont occurrences are shown for the expanded site 3. Question marks refer to either uncertainties of redox interpretation or age correlations. Chan., Changsingian; Dien., Dienerian; Gr., Griesbachian; Smith., Smithian; Wo., Wordian; Wu., Wuchiapingian.

Fe-speciation has traditionally been applied to fine-grained siliciclastic rocks, and 60% of the samples we utilize here for Fe-speciation are siliciclastics. In addition, a recent assessment has demonstrated the robust nature of the proxy in carbonate-rich sediments, providing Fe_T_ is >0.5 wt% (ref. 35). When Fe_T_ is <0.5 wt%, carbonate samples have greater potential to be spuriously enriched in Fe_HR_ from processes other than the water column enrichments that arise under anoxic conditions. This potential effect is apparent in our samples from the distribution of Fe_HR_/Fe_T_ against Fe_T_ where samples with Fe_T_ <0.5 wt% almost exclusively give Fe_HR_/Fe_T_ ratios>0.38 (Fig. 2), despite independent evidence of oxygenation from bioturbation and benthic fauna associated with some samples (Supplementary Figs 1–7). Hence, in the present study, we do not utilize Fe-speciation or Fe/Al data for samples with <0.5 wt% Fe_T_. Instead, for these samples, we rely on palaeontological information to identify whether oxic water column conditions were likely prevalent. In support of our approach, interbedded carbonates and siliciclastics give consistent redox interpretations in all cases (Figs 2 and 3; Supplementary Figs 1–7).

The two indicators of anoxia generally show consistent results (Fig. 3; Supplementary Figs 1–7; Supplementary Tables 1–7); however, across some intervals, Fe/Al ratios are relatively low, which contrasts with clear Fe_HR_/Fe_T_ enrichments (starred intervals, Fig. 3). For example, this pattern is seen in the Late Permian and mid-Dienerian of the deep basin, around the anoxic episode at the EI in the mid-slope, at the end-Dienerian in the platform marls and shales, and in both slope sites from the mid-to-late Smithian in laminated carbonates. These data are unlikely to reflect an addition of Fe_HR_ during deep burial dolomitization, as such samples were avoided in this study (Supplementary Note 4). It is also unlikely that these limited samples reflect deposition under suboxic conditions or Fe_HR_ dilution due to high sedimentation rates, as this would affect both Fe/Al and Fe_HR_/Fe_T_ ratios in the same manner. However, Fe/Al tends to suffer from greater inherent variability due to local compositional variability in the source rocks, and therefore using a local oxic baseline is recommended where possible^18^,^33^. A major transgression during the mid-to-late Permian created extensive carbonate deposition landward of our sites^20^. Erosion during the Early Triassic then penetrated down to the mid-Permian deposits only^20^. Thus, the hinterland lithogenic source of Fe for the Arabian Margin was primarily limited to low Fe carbonate sediments. Samples with elevated Fe_HR_/Fe_T_ and relatively low (or borderline) Fe/Al ratios may then indeed reflect anoxic conditions, but Fe enrichments were either not sufficient to significantly increase Fe/Al ratios above the natural variability, or the regional oxic Fe/Al baseline was lower than the global average.

### Water column redox conditions

Anoxic water column deposition is identified by elevated Fe_HR_/Fe_T_ and Fe/Al ratios at three main time intervals across the Arabian Margin transect: the Changsingian and overlying EI, and the Dienerian–Smithian and Smithian–Spathian boundary intervals. There is strong evidence for more persistent anoxia, from consistently elevated Fe_HR_/Fe_T_ and Fe/Al ratios, in the distal slope setting for much of the Early Triassic, while significant spatial and temporal variability is seen elsewhere in the basin. In addition, low Fe_py_/Fe_HR_ ratios throughout the succession (Fig. 3) demonstrate that anoxic intervals were ferruginous, with no evidence for the pyrite enrichments that would be prevalent under euxinic conditions.

In the Late Permian, high Fe_HR_/Fe_T_ and low Fe_Py_/Fe_HR_ ratios are first recorded in deep basin shales during the Late Capitanian to Early Changsingian (site 6, Fig. 3; Supplementary Fig. 7) and persisted until the end of the EI. The lowest samples here are radiolarian shales, a lithology that has not been tested rigorously for Fe-based redox proxies and thus the possibility of anoxia in the Capitanian to Wuchiapingian cannot be confirmed. Anoxic conditions appear only intermittently in mid-slope settings in the latest Changhsingian (*C. changxingensis* zone), where Fe_HR_-enriched shales alternate with bioturbated limestones. Anoxia was then persistent throughout the EI until at least the *I. isarcica* zone (site 3, Fig. 3, Supplementary Fig 2 and 3), although the onset of extinction is not preserved due to a minor unconformity.

In the Griesbachian (within the *I*. *isarcica* zone), Fe-speciation and Fe/Al data suggest that oxygenated conditions were present in the deepest basin (Fig. 3). At this time, the middle slope sediments show intense bioturbation (75–80 m, Supplementary Fig. 3), while other Arabian platform areas (site 2) display a highly diverse assemblage of crinoids, gastropods and bivalves that are not seen outside of the Neo-Tethys until the Spathian^36^. Together, this suggests a period of stable water column oxygenation for the entire region, when the Arabian Margin appears to have acted as a site for rapid biotic recovery^36^.

Records from the distal slope setting (site 4) begin in the Dienerian and suggest largely persistent anoxic ferruginous conditions (Fig. 3), with high Fe_HR_/Fe_T_ ratios and no evidence of bioturbation throughout the Early Triassic. During the Dienerian, the mid-slope sediments record a single Fe_HR_ enrichment in laminated sediments followed by a restricted interval of vermicular limestones that ended around the Dienerian/Smithian boundary. The limited degree of bioturbation preserved by vermicular limestones indicates an unstable and rapidly fluctuating environment^37^. Together, these data suggest an environment that was predominantly anoxic on the distal slope, from at least the Dienerian, but which fluctuated between anoxic and oxic elsewhere (Fig. 3). The low Fe_T_ sediments of the platform show bioturbation until the end-Dienerian, suggesting this setting remained oxygenated. This oxygenated period ended with an increase in Fe_HR_/Fe_T,_ with low Fe_Py_/Fe_HR_, indicating a distinct period of ferruginous conditions in shallow waters; the only occurrence of this in the Arabian Margin record. Unfortunately, the mid-slope section contains minor unconformities and the lower sample resolution limits correlation of this anoxic pulse across the margin.

During the Smithian, the slope records show subtle variability in redox conditions. For the mid-slope in the early to mid Smithian there is a single Fe_HR_-enriched laminated carbonate (Fig. 3; Supplementary Figs 2 and 3) followed by low Fe_T_ samples with rare bioturbation. Corresponding distal-slope sediments also have low Fe_T_ (56–78 m; Supplementary Fig. 4), precluding Fe proxy evaluation^35^, but nevertheless bioturbation is absent. A number of ferruginous samples (without bioturbation) are then present in the mid-slope during the mid-Smithian, followed again by low Fe_T_ samples into the late Smithian (0–210 m; Supplementary Fig. 3). These low Fe_T_ samples correspond to an increased bioturbation frequency and presence of small foraminifera. Interestingly, however, ichnodiversity remained low and was restricted to shallow tiers within the sediment^23^ (Fig. 3), suggesting only temporary periods of oxygenation. Oxic deposition is then recorded briefly by low Fe_HR_/Fe_T_ and Fe/Al ratios in shales of the latest-Smithian (220–230 m; Supplementary Fig. 3). Integrating Fe-speciation data with palaeontological observations across these two sites suggest rapidly fluctuating oxygenation of the mid-slope. This ceased abruptly near the Smithian/Spathian boundary, where both Fe-speciation and Fe/Al show clear enrichments, recording a return to anoxic conditions in the mid-slope, while anoxia continued in the distal slope. Conversely, platform records show low Fe_T_ values at the Smithian/Spathian boundary (Fig. 3; Supplementary Fig. 1), and breccia deposits in the mid-slope contain disarticulated crinoid ossicles, together suggesting that oxygenated conditions were present, at least locally, in shallower waters. This brief anoxic episode was followed by a return of oxygenation in the mid-slope during the Spathian, with Fe_HR_/Fe_T_ and Fe/Al returning to lower values.

The deep basin is more difficult to interpret as age uncertainties make correlation of these events problematic, but the record suggests fluctuating conditions throughout the Dienerian to Smithian, similar to the mid-slope site. The seamount section (site 5; Fig. 3) gives unique insight into the evolution of water column anoxia by capturing the mid-depth water column signature. The unusual carbonate fabrics preserved here during the Smithian (abiotic calcite spar, for example, *Frutexites* and *Stromatactis*) are indicators of atypical ocean chemistry during the Early Triassic^25^. It has been suggested that the presence of these fabrics at this isolated locality may be linked to its position within a fluctuating chemocline and resulting variability in calcium carbonate saturation state^25^. Low Fe_T_ contents of these carbonate samples (Fig. 3; Supplementary Fig. 6) prevent interpretation of Fe-speciation data and thus the direct presence of water column anoxia cannot be confirmed. However, the close proximity of the seamount to a fluctuating chemocline is consistent with dynamic anoxia recorded in the adjacent slope environment.

### Sulfur isotopes

**Figure 4:**
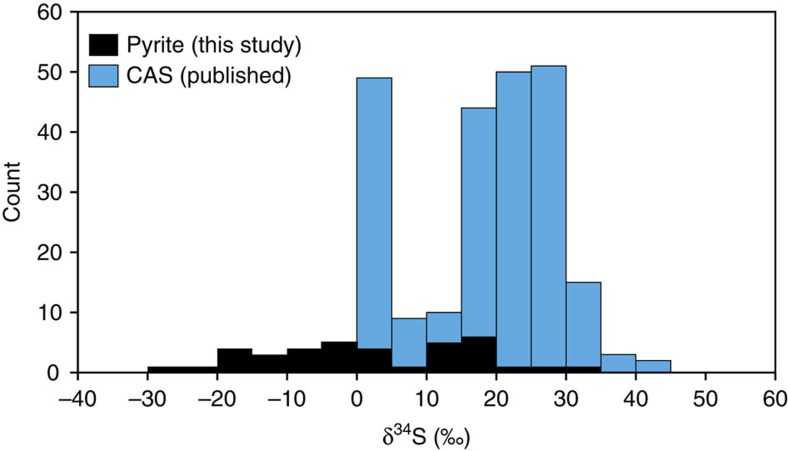
S-isotope systematics. Comparison of new δ^34^S_py_ data with published values of δ^34^S_seawater_ from Late Permian and Early Triassic CAS records^40^,^41^. The overlap between the two δ^34^S records supports the inference of ferruginous conditions from Fe-speciation and suggests low background sulfate concentrations.

Our δ^34^S_pyrite_ data range from −27.3‰ to +32.5‰ for anoxic samples (Fig. 4), with the data skewed towards heavier values. Under euxinic conditions, a narrow range in δ^34^S_py_ is often found in modern and ancient environments^38^, due to sulfide production in a well-mixed water column. In contrast, diagenetic pyrite formation generally exhibits a wider range in δ^34^S_py_ consistent with the magnitude of fractionations commonly found through bacterial sulfate reduction during early diagenesis^39^. In comparison, isotopic records of Early Triassic seawater sulfate (from carbonate-associated sulfur; CAS) are highly positive (Fig. 4), ranging between −0.7 and +44.1‰ (refs 40, 41). The considerable overlap between the seawater sulfate and pyrite sulfur isotopic records is consistent with a relatively low seawater sulfate reservoir^38^,^42^.

## Discussion

Previous studies have identified both deeper water (using framboid size distributions) and photic zone (using biomarkers) euxinia periodically through the Late Permian and Early Triassic^4^,^6^,^43^. Euxinia appears particularly prevalent in the Palaeo-Tethys and the Panthallassic Oceans, with no conclusive reports for the Central Neo-Tethys. By contrast, all anoxic data presented here record low Fe_Py_/Fe_HR_ ratios, suggesting a predominantly ferruginous rather than euxinic water column. Fe-shuttling within a suboxic oxygen minimum zone (OMZ) could potentially create similar local enrichments in Fe-oxides^44^, resulting in high Fe_HR_/Fe_T_, Fe/Al and low Fe_Py_/Fe_HR_ ratios. In this scenario, the reductive mobilization of Fe (II) under suboxic conditions may lead to depletion of Fe in the sediment underlying the OMZ itself^44^ (that is, Fe/Al below the lithogenic baseline combined with low Fe_HR_/Fe_T_ ratios). The mobilized Fe would then be transported within the suboxic OMZ and sequestered at the lower oxycline, leading to an Fe_ox_ enrichment in bioturbated sediments below the OMZ^44^. This is not seen in our Permo-Triassic Arabian Margin record where the greatest enrichments of Fe_HR_/Fe_T_ are in the distal slope environment, where bioturbation is completely absent. Also, if the distal slope site were suboxic, we would expect depletions of Fe_HR_, not enrichments, as these sediments would act as an Fe(II) source. Our data are therefore more consistent with distal slope anoxic ferruginous conditions, which expanded and contracted through time, leading to occasional Fe enrichments in shallower and deeper settings.

Our Fe-speciation data suggest an environment characterized by low pyrite burial, and sulfur isotopes, both from the δ^34^S_py_ presented here and previously published δ^34^S_CAS_ data^40^,^41^,^45^, are consistent with low sulfate concentrations in the water column during the PTB and the Early Triassic. The increase in bedrock weathering intensity in the Early Triassic^46^,^47^ would have liberated some sulfate from the continent, implying that the low-sulfate ocean state was a result of high sulfate removal, potentially through substantial pyrite deposition under euxinic conditions that are seen elsewhere^4^,^45^, augmented via the large-scale deposition of marine evaporites containing disproportionately large amounts of sulfate in the late Permian and earliest Triassic^48^. In addition to low ocean sulfate concentrations, the proximity of the Arabian Margin to the open ocean, and a limited flux of organic carbon, may have prevented conditions becoming sufficiently reducing for sulfide to accumulate in the water column, which is consistent with the absence of organic-rich black shales in the Arabian sites (Supplementary Figs 1–7).

The precarious balance between ferruginous and euxinic chemical states is dictated by the relative oceanic input fluxes of Fe_HR_ and sulfate^13^. Driven by extreme temperatures^49^, silicate weathering is thought to have intensified during this time^46^,^50^, likely leading to increased release of Fe_HR_ from parent silicate minerals^28^ and thus a disproportionate increase in the flux of Fe_HR_ relative to sulfate. In addition, under the arid climate of the Permo-Triassic, dust production, and therefore the aeolian Fe flux, would also be higher^51^, which may have been further exacerbated at the PTB by the loss of terrestrial vegetation that led to soil destabilization^52^. On a global scale, these continental sources represent the greatest potential flux of Fe_HR_^13^,^28^; however, on a more regional scale, hydrothermal inputs could also be important. There is indeed evidence that the Arabian Margin was in close proximity to a hydrothermal source of Fe(II), both from mid-Permian basalts underlying the deep basin sediments^24^ and from a spreading centre in the Neo-Tethys^53^. Temporally limited deep basin anoxia, identified here, provides a plausible mechanism for the transport of this Fe(II) during the Late Permian and occasionally in the Early Triassic. Regardless of the Fe source, anoxic conditions would still be required to explain the observed Fe_HR_ enrichments across the shelf-to-basin transect. Thus there are numerous reasons why the Fe_HR_-to-sulfate ratio of the oceans may have been skewed in favour of ferruginous conditions and we expect a combination of mechanisms to be important.

**Figure 5:**
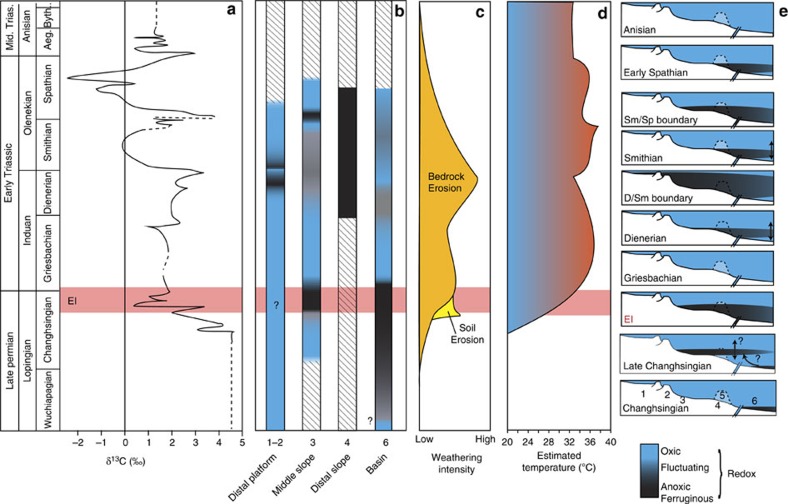
Compilation of global records compared to the Arabian Margin redox record. (**a**) Regional δ^13^C curve from the Musandam Mountains, UAE that correlates well with global records and especially the sections presented here^21^. (**b**) Summary of redox interpretations from Fig. 2. Hashed areas are not preserved in the Arabian Margin. (**c**) Global weathering rates^46^ demonstrating an increase in soil erosion and silicate weathering, approximately coincident with the development of slope anoxia. (**d**) Equatorial temperature changes^49^. (**e**) Schematic diagram illustrating the redox dynamics of the Arabian Margin for the end-Permian and Early Triassic based on the data presented here. Arrows represent upwelling and expansions of an anoxic ferruginous wedge. Extinction interval (EI) shown as red horizontal box.

The presence of deep water anoxia on the Late Permian Arabian Margin (Fig. 3) suggests that the oceans were already primed for a later expansion of anoxic conditions and subsequent environmental crisis. This could represent the interplay of poor oxygenation due to sluggish circulation and high oxygen consumption during organic matter decay in a nutrient rich, highly productive Late Permian ocean^54^,^55^. Moving upwards to the EI itself, the mid-slope anoxic episode recorded here corresponds to the negative PTB carbon isotope excursion, a record that is dominated by a flux of isotopically light carbon to the atmosphere from a combination of methane, volcanism or a decrease in the removal of light carbon resulting from the terrestrial mass mortality^56^,^57^. This observation is consistent with numerous sections worldwide^9^,^46^,^58^,^59^, whereby slope and shallow water anoxia is thought to have been driven by nutrient loading from either a weathering pulse, increased soil erosion at the PTB^9^,^46^,^58^ (Fig. 5), or the upwelling of deep water anoxia/euxinia^60^.

During the protracted recovery period, our new data are consistent with previous suggestions for the episodic nature of anoxic events^4^,^10^,^11^. For the first time, however, we can also identify that the maximum extent of anoxia across the Arabian Margin is consistently associated with the globally recorded positive carbon isotope excursions (CIEs; Fig. 5) at both the Dienerian/Smithian boundary (shallow water anoxia) and the Smithian/Spathian boundary (mid-slope anoxia). Unfortunately, our records do not extend to the positive CIE of the Early/Middle Triassic boundary, but we would predict a further redevelopment of anoxia at this point. Although some published records show this relationship^11^,^43^,^61^,^62^, data for the Early Triassic provide mixed observations, often with no consistent pattern of anoxia^4^,^11^ or correlation with the negative CIEs^10^,^59^. The relationship between redox evolution and the δ^13^C record has implications for the driving mechanisms of anoxia and the role of internal feedback versus external forcing processes. Some of the differences in redox records will reflect palaeogeography and palaeodepth variations, where only a snapshot of the complex redox structure has been seen previously, in comparison with the vertical resolution afforded by the Arabian Margin sites. We also expect the lack of global consistency to be due to limitations in the redox proxies previously used, as they have been unable to identify anoxic ferruginous conditions.

The end-Dienerian event coincides with peak Early Triassic sedimentation rates^47^ (Fig. 5c), suggesting that high weathering rates and nutrient influxes could have driven the water column anoxic. In contrast, the Smithian/Spathian boundary event occurred during an apparent decrease in global weathering rates^47^. The event is, however, closely associated with decreasing temperatures after the Late Smithian equatorial thermal maximum^49^ (Fig. 4). Greater ocean stagnation may be expected at the thermal maximum, and although the reconstructed temperature drop is relatively modest, it may have driven a reinvigoration of circulation that stimulated productivity or led to upwelling of anoxic waters.

The periodic development of anoxic ferruginous conditions on the Arabian Margin has major implications for the mass extinction event itself, and for biogeochemical cycling in general across this interval. Sulfide is highly toxic to almost all eukaryotes at micromolar concentrations^2^ and is therefore a kill mechanism in its own right, but the lack of euxinia suggests that sulfide was not responsible for the extinction in this area of the Neo-Tethys, a conclusion that has also recently been suggested for parts of the Palaeo-Tethys^63^. Organisms can adapt to low oxygen conditions (for example, the Lilliput effect^64^), and low oxygen tolerant species are selectively seen in the Early Triassic fossil record^2^,^36^. However, periods of regionally stable oxygenation appear to facilitate exceptional biotic recovery during the Griesbachian on the Arabian Margin. Thus, the rapid and repeated development of anoxic, but non sulfidic, conditions must have placed a major restriction on the biota in this area.

The precise chemical state of the water column also has major implications for the biogeochemical cycling of nutrients such as P and N. Current carbon cycle models for this time period require the inclusion of a positive-feedback mechanism^56^,^65^, whereby under anoxic conditions P is recycled back into the water column, thereby stimulating further productivity to drive the total organic carbon depositional flux and maintain anoxia^66^. Indeed, the P-feedback mechanism appears to be an essential part of driving global positive CIEs during OAEs^67^. This, however, is less applicable under ferruginous conditions, whereby P is more likely to be sequestered in the sediment during uptake by Fe-(oxyhydr)oxide minerals^68^. The development of euxinia is also closely tied to the N cycle, being dependent on the mode of nitrogen supply to the photic zone^16^. The behaviour of P and N under these different chemical states has great potential to influence global biogeochemical cycles^14^,^16^, the longevity of anoxic events^15^ and therefore the timing of biotic recovery after the end-Permian mass extinction. These factors suggest that further evaluation of the global extent of ferruginous conditions is required to fully understand biogeochemical controls on anoxia and life across the end-Permian mass extinction and recovery.

In summary, episodes of locally anoxic ferruginous conditions identified across the Arabian Margin appear to be manifested as increases in the spatial extent and temporal stability of anoxia, corresponding to changes in the global carbon cycle driven by global mechanisms. Biotic recovery in the Neo-Tethys was rapid during longer periods of oxygenation, confirming that the Early Triassic represents a period of repeated environmental perturbations on a global scale, which had a highly variable manifestation. The exact regional redox expression of anoxia, in terms of structure and chemical state, was likely dictated by local productivity, bathymetry and circulation differences between the Early Triassic oceans, as well as the local supply of Fe_HR_ and regional sulfate drawdown^13^. The implications of the dominance of ferruginous conditions during this complex event require further investigation, but these data establish an important link between the precise chemical state of anoxic events and both carbon cycle stability and biotic resilience.

## Methods

### Fe-speciation and Fe/Al

Weathered surfaces were removed from rock samples using a diamond-tipped saw. Samples were then fragmented and powdered using a jaw crusher and Tema mill with Tungsten Carbide barrel. Fe-speciation extractions were performed according to the calibrated extraction procedure^12^, whereby Fe_Carb_ was extracted with Na-acetate at pH 4.5 and 50 °C for 48 h, Fe_Ox_ was extracted via Na-dithionite at pH 4.8 for 2 h, and Fe_Mag_ was extracted with ammonium oxalate for 6 h. Fe_T_ extractions were performed on ashed samples (8 h at 550 °C) using HNO_3_–HF–HClO_4_. Boric acid was used to prevent the formation of Al complexes, allowing Fe/Al to be determined on the same extractions. All Fe concentrations were measured via atomic absorption spectrometry and replicate extractions gave a relative standard deviation (RSD) of <5% for all steps. Pyrite Fe was determined stoichiometrically by weight from precipitated Ag_2_S after chromous chloride distillation^69^. Fe/Al ratios were measured on the total extracts using optical emission spectrometry, with replicate extractions giving a RSD of <1.7%.

### Pyrite sulfur isotopes

Pyrite S-isotope compositions (δ^34^S_pyrite_) are also used to give further insight into sulfur cycling and to provide additional support for water column redox interpretations. δ^34^S_pyrite_ analyses were performed on the Ag_2_S precipitate produced from the chromous chloride distillation, using an Elementar Pyrocube coupled to an Isoprime stable isotope mass spectrometer.

### Carbon isotopes

All measurements were carried out on a Thermo Finnigan GASBENCH II linked online to a Thermo Finnigan DELTA V 94 isotope ratio mass spectrometer at the Museum fuer Naturkunde Berlin, Germany. Isotope ratios are reported in δ-notation in (‰) relative to the Vienna Peedee Belemnite. The analytical reproducibility of δ^13^C_carb_ and δ^18^O values is each generally better than ±0.2‰ (2 s.d.)

### Data availability

All data are provided in the Supplementary Information.

## Additional information

**How to cite this article:** Clarkson, M. O. *et al*. Dynamic anoxic ferruginous conditions during the end-Permian mass extinction and recovery. *Nat. Commun.* 7:12236 doi: 10.1038/ncomms12236 (2016).

## Supplementary Material

Supplementary Information Supplementary Figures 1-7, Supplementary Tables 1-7, Supplementary Notes 1-4 and Supplementary References.

## Figures and Tables

**Figure 1:**
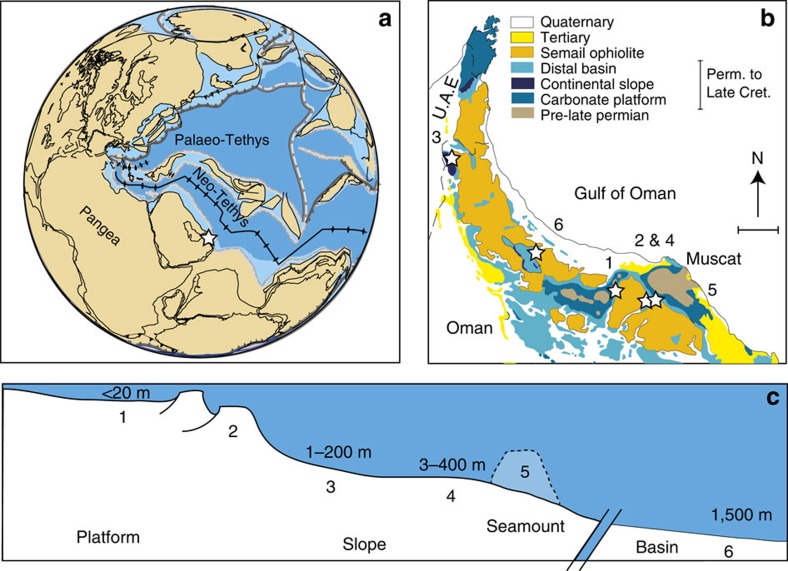
Geological information. (**a**) Late Permian palaeogeographic reconstruction^53^,^70^ with the location of the Arabian Margin marked. (**b**) Simplified geological map of Oman and UAE^70^ showing site localities; scale bar, 50 km; (1) Saiq Plateau; platform, (2) Wasit Block; distal platform; (3) Sumeini; middle slope, (4) Wadi Wasit South and Radio Tower; distal slope, (5) Ba'id; seamount, (6) Buday'ah; deep basin. (**c**) Schematic basin cross-section for the Dienerian (not to scale) showing estimated palaeo-depths (based on facies) and site numbers as in **b**. See Supplementary Note 1 for detailed site descriptions.
