# Supplementary material for: Dynamic anoxic ferruginous conditions during the end-Permian mass extinction and recovery
Source: Nat Commun. 2016 Jul 19;7:12236. doi: 10.1038/ncomms12236 (PMC4960316; doi:10.1038/ncomms12236)
Supplement: Supplementary Information — Supplementary Figures 1-7, Supplementary Tables 1-7, Supplementary Notes 1-4 and Supplementary References. [file ncomms12236-s1.pdf]

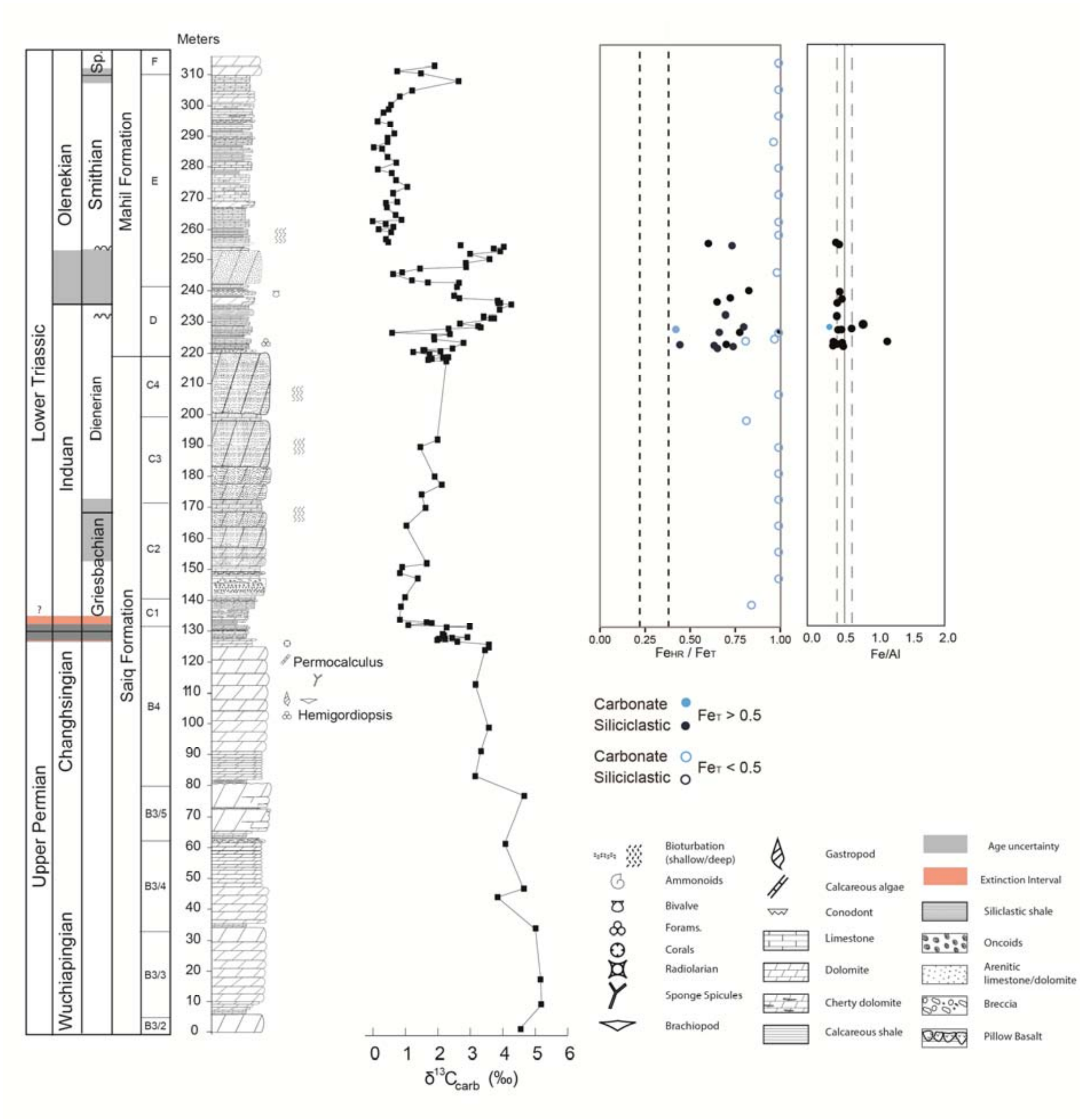

Supplementary Fig. 1 – Distal Platform Site 1, Saiq Plateau.

Stratigraphy and $\delta^{13}C$ from the equivalent Wadi Sahtan section[1] that is well-correlated with the Saiq Plateau. Includes key for all sections.

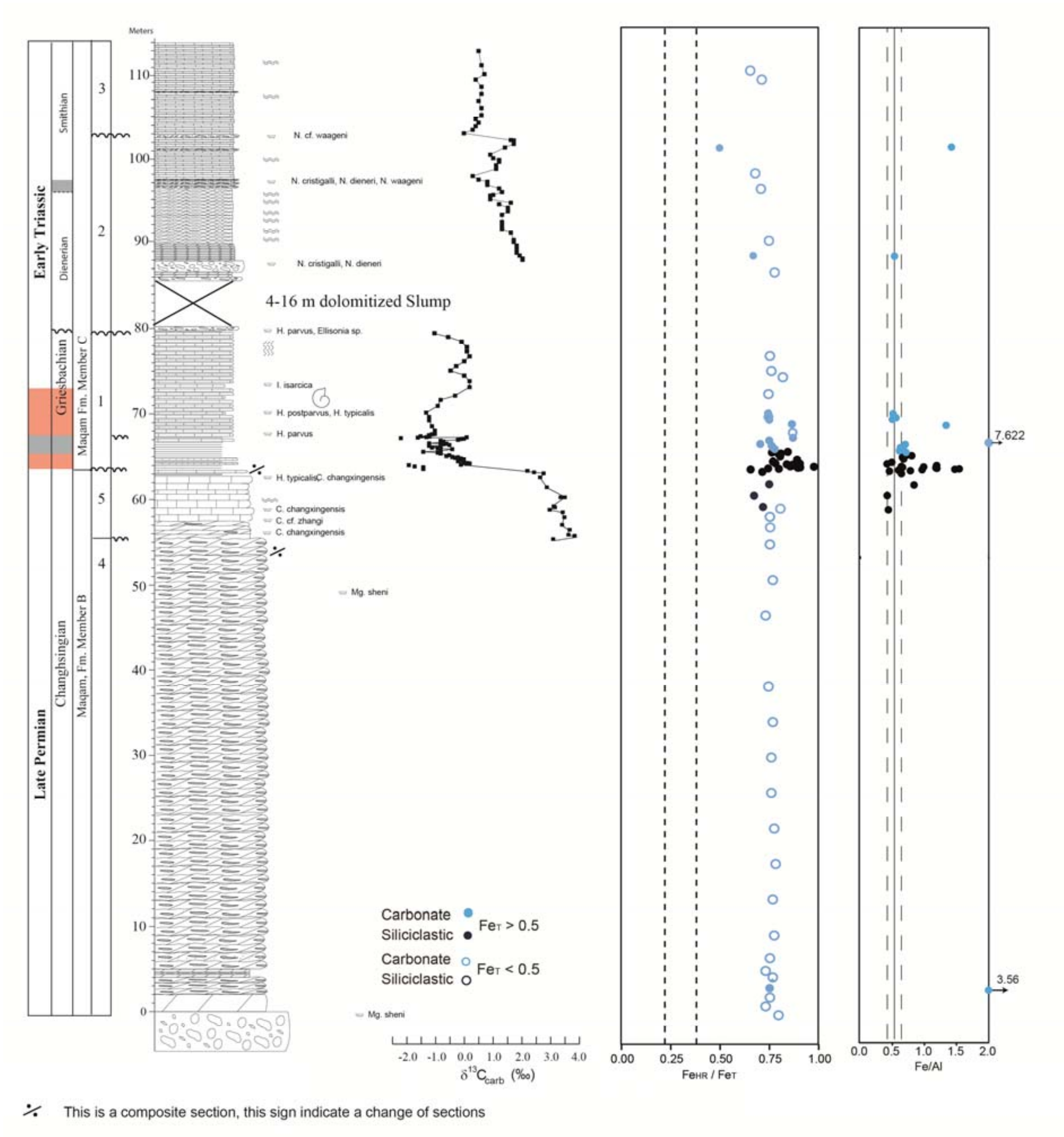

Supplementary Fig. 2- Mid-slope Site 3. Sumeini Group, Wadi Maqam.

Stratigraphy and $\delta^{13}C$ from ref. [1]

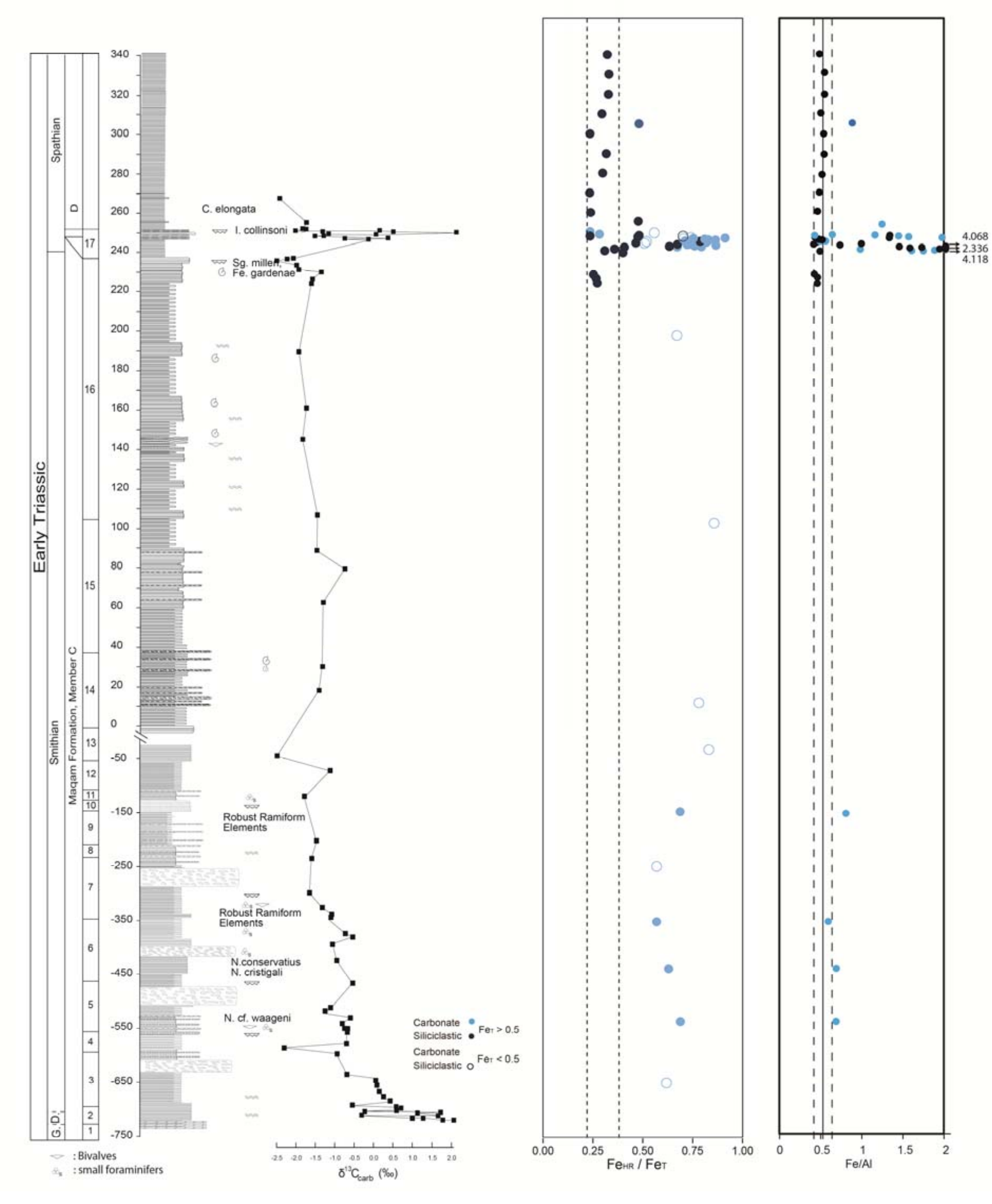

Supplementary Fig. 3 – Mid-slope Site 3 continued. Sumeini Group, Wadi Shuyab. *Stratigraphy and $\delta^{13}C$ from ref [1]. Note change in scale below 0 m due to higher sedimentation rates in the Smithian.*

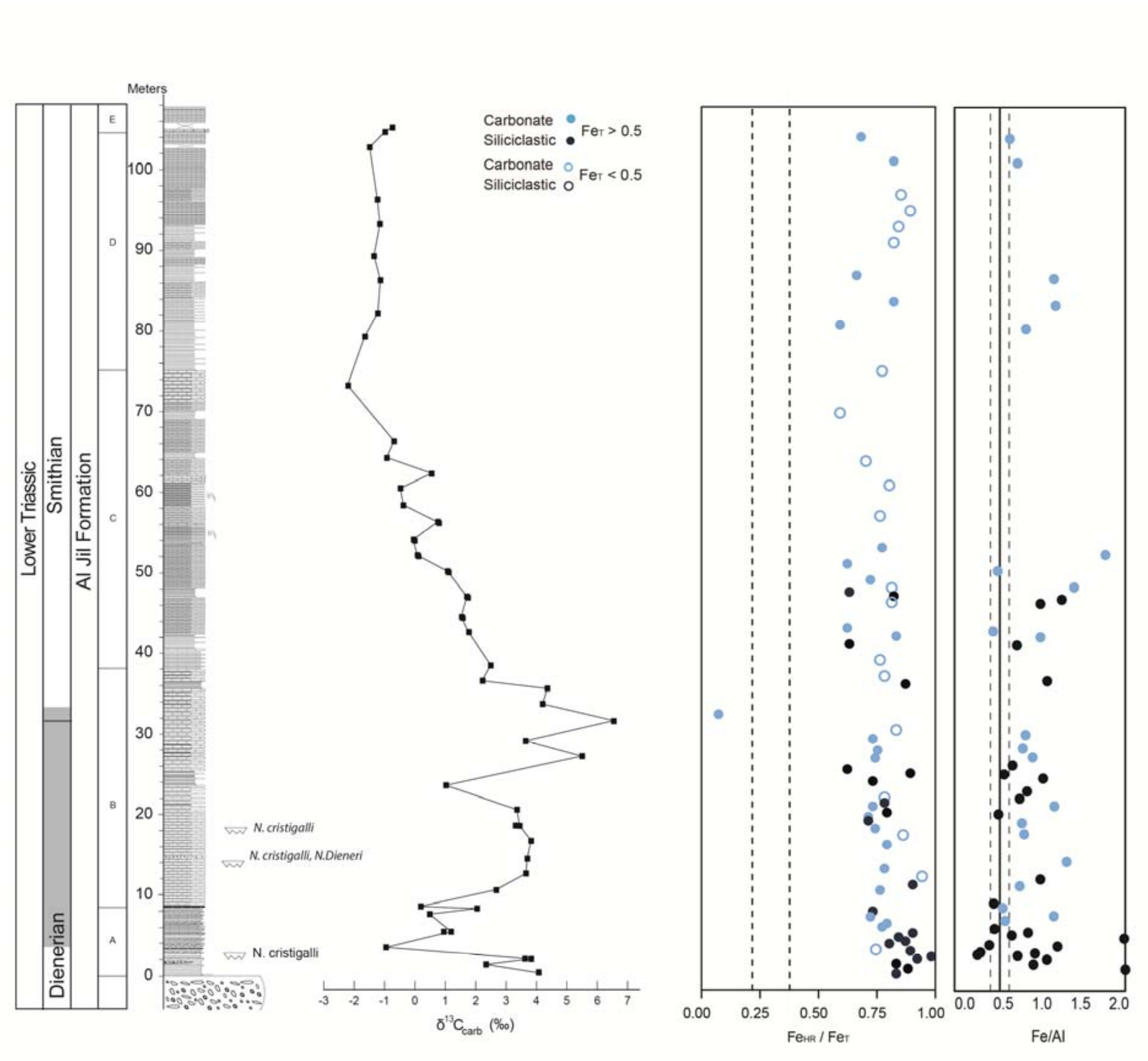

Supplementary Fig. 4 – Distal slope Site 4. Wadi Wasit South.

Stratigraphy and $\delta^{13}C$ from ref [1].

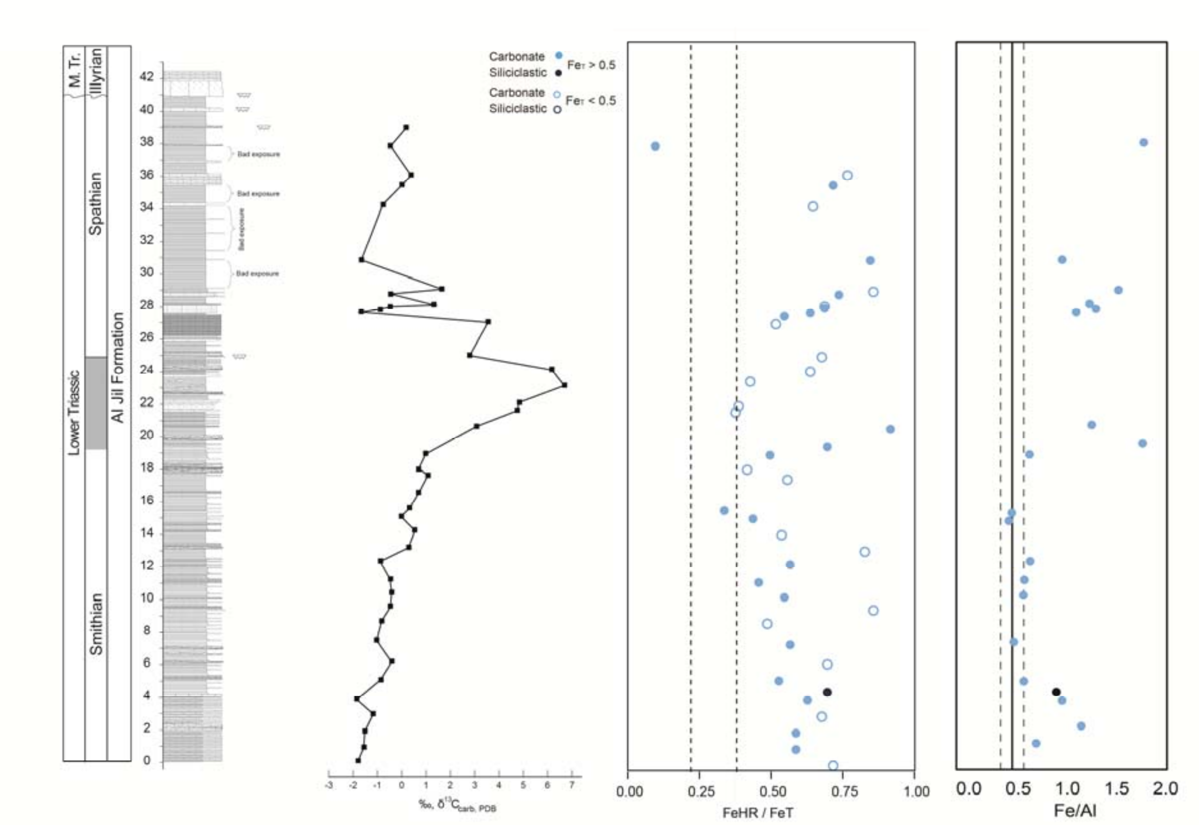

Supplementary Fig. 5 – Distal slope Site 4 continued, Radio Tower.

Stratigraphy and $\delta^{13}C$ (this study).

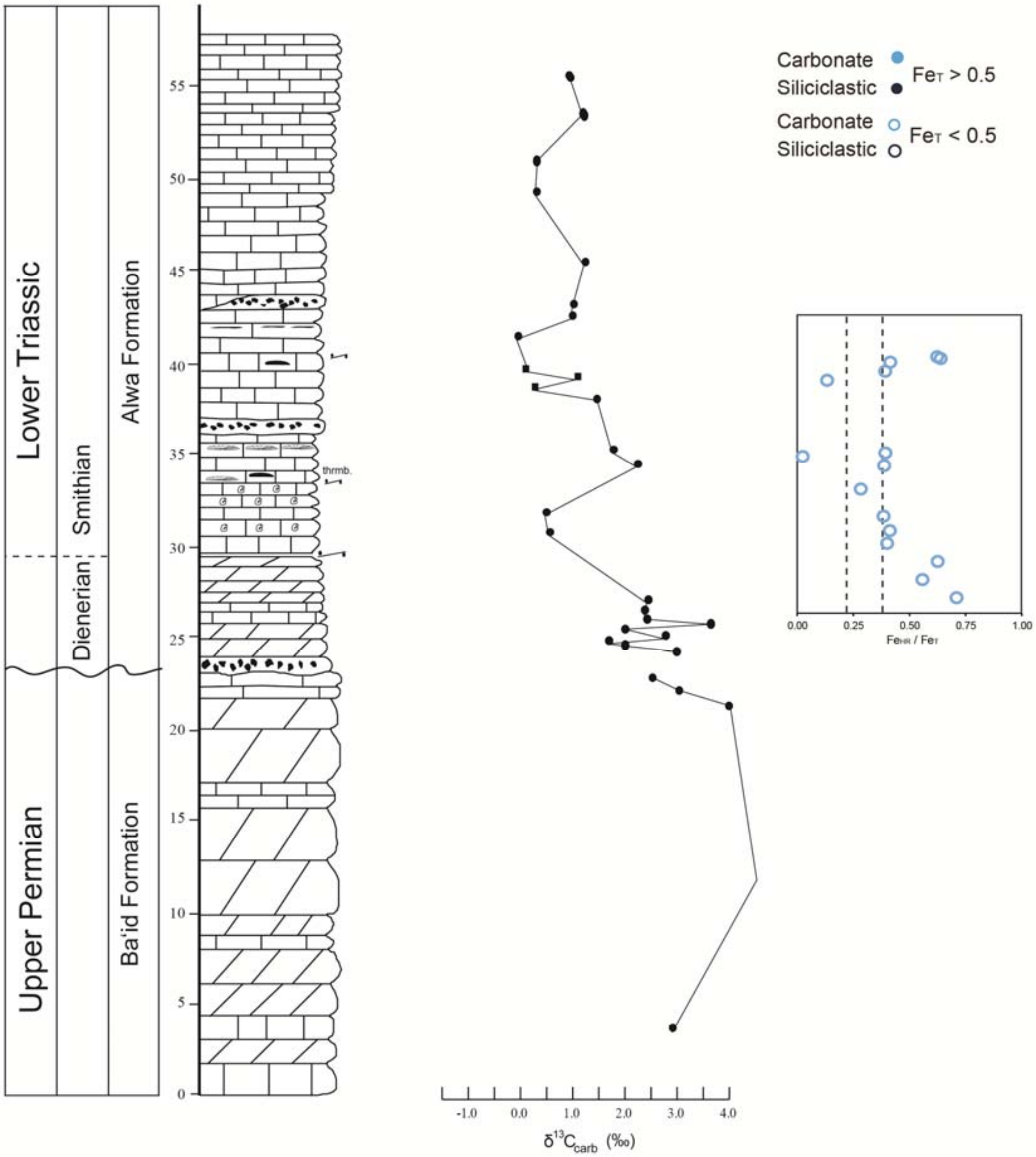

Supplementary Fig. 6 – Isolated Seamount Site 5, Baid.

Stratigraphy and $\delta^{13}C$ *[ref. 2]. A possible anoxic event is recorded in elevated* Fe_{HR}/Fe_T *ratios at the end of the Dienerian (which corresponds with anoxia recorded at the platform site). However, low* Fe_T *values preclude the use of Fe-speciation for this site. The carbonate fabrics during this interval are, however, consistent with the reconstructed anoxic wedge scenario.*

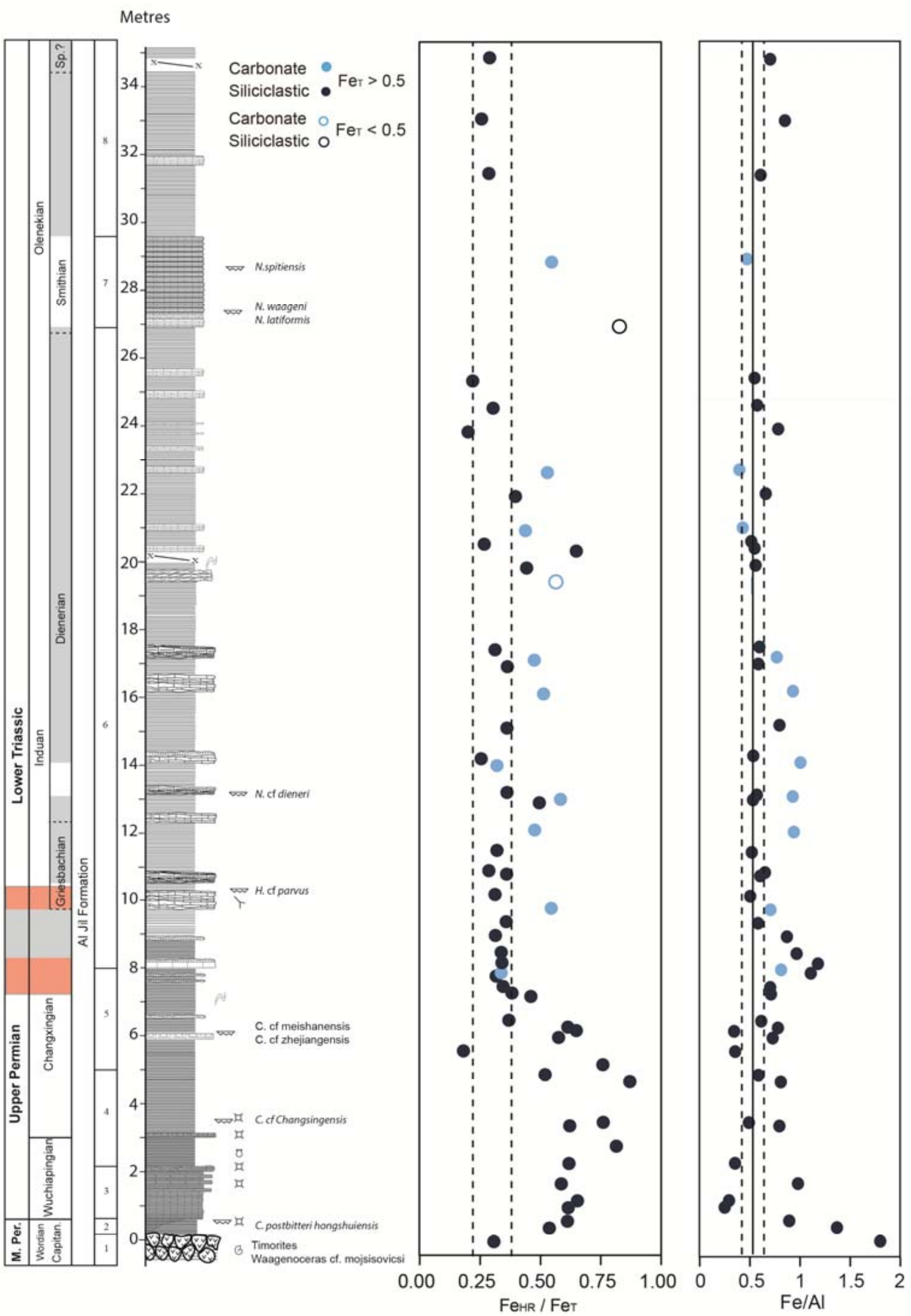

Supplementary Fig 7: Deep Basin Site 6, Buday'ah.

Stratigraphy modified from [3].

Supplementary Table 1: Site 1, Distal Platform, Saiq Plateau. Heights refer to Supplementary Fig. 1. Fe data in wt%.

Height (m)	Locality	Number	Lith	Fe_T	Fe_{Carb}	Fe_{Ox}	Fe_{Mag}	Fe_{Py}	Fe_{HR}/Fe_T	Fe_{Py}/Fe_{HR}	Fe/Al
140	SQD	1	Carbonate	0.090	0.018	0.041	0.012	0.002	0.840	0.033	
145	SQD	2	Carbonate	0.060	0.018	0.043	0.016	0.002	1.270	0.023	
155	SQD	3	Carbonate	0.050	0.015	0.024	0.009	0.002	1.020	0.031	
165	SQD	4	Carbonate	0.030	0.013	0.018	0.010	0.002	1.430	0.047	
170	SQD	5	Carbonate	0.050	0.021	0.017	0.010	0.001	1.080	0.048	
180	SQD	6	Carbonate	0.030	0.020	0.016	0.014	0.002	1.760	0.011	
190	SQD	7	Carbonate	0.040	0.016	0.021	0.013	0.001	1.340	0.040	
200	SQD	8	Carbonate	0.040	0.000	0.013	0.017	0.001	0.810	0.015	
210	SQD	9	Carbonate	0.030	0.040	0.014	0.017	0.000	2.140	0.022	
220	SAIQ1	1	Carbonate	0.516	0.134	0.259	0.000	0.023	0.805	0.055	0.513
220.25	SAIQ1	2	Siliciclastic	1.362	0.043	0.824	0.012	0.013	0.656	0.018	0.360
220.75	SAIQ1	3	Carbonate	0.455	0.140	0.290	0.000	0.011	0.976	0.034	
220.85	SAIQ1	4	Siliciclastic	0.816	0.068	0.518	0.006	0.016	0.745	0.026	0.423
221.35	SAIQ1	5	Siliciclastic	3.165	0.036	1.912	0.061	0.011	0.638	0.006	0.497
221.75	SAIQ1	6	Siliciclastic	0.967	0.035	0.630	0.011	0.007	0.706	0.010	0.366
221.85	SAIQ1	7	Siliciclastic	3.344	0.007	1.452	0.041	0.002	0.449	0.001	1.168
224.35	SAIQ1	8	Carbonate	0.147	0.099	0.046	0.000	0.004	1.015	0.035	
226.25	SAIQ1	9	Siliciclastic	1.551	0.632	0.065	0.003	0.508	0.780	0.422	0.436
226.35	SAIQ1	10	Siliciclastic	2.253	0.071	1.396	0.026	0.010	0.668	0.008	0.486
226.75	SAIQ1	11	Siliciclastic	0.977	0.155	0.608	0.000	0.220	1.007	0.225	0.637
227.35	SAIQ1	12	Carbonate	2.749	0.000	1.127	0.047	0.001	0.427	0.001	0.306
228.35	SAIQ1	13	Siliciclastic	0.762	0.164	0.349	0.005	0.086	0.802	0.152	0.808
231.35	SAIQ1	14	Siliciclastic	2.439	0.107	1.522	0.027	0.053	0.701	0.032	0.417
234.75	SAIQ1	15	Siliciclastic	1.692	0.210	1.045	0.009	<0.001	0.655	0.000	0.423
236.25	SAIQ1	16	Siliciclastic	1.261	0.290	0.673	0.000	<0.001	0.728	0.000	0.493
239	SAIQ1	17	Siliciclastic	1.538	0.272	0.775	0.006	0.226	0.831	0.177	0.460
241.5	SQA	34	Siliciclastic	0.100	0.034	0.050	0.010	0.003	0.990	0.032	
254.5	SAIQ1	18	Siliciclastic	0.876	0.079	0.551	0.003	0.015	0.739	0.023	0.452
255.25	SAIQ1	19	Siliciclastic	1.235	0.050	0.726	0.005	<0.001	0.606	0.000	0.404

256.25	SAIQ1	20	Carbonate	0.126	0.146	0.225	0.000	<0.001	2.874	0.000
260	SQB	1	Carbonate	0.110	0.054	0.040	0.012	0.006	1.040	0.053
270	SAIQ1	21	Carbonate	0.412	0.108	0.188	0.000	0.006	0.730	0.019
280	SQB	5	Carbonate	0.160	0.106	0.042	0.017	0.003	1.070	0.016
285	SQB	6	Carbonate	0.100	0.063	0.023	0.011	0.002	1.000	0.017
295	SQB	7	Carbonate	0.190	0.089	0.078	0.020	0.001	0.970	0.005
305	SQB	8	Carbonate	0.160	0.103	0.042	0.014	0.003	1.040	0.019
310	SQB	9	Carbonate	0.080	0.034	0.051	0.010	0.001	1.150	0.015
315	SQB	11	Carbonate	0.060	0.042	0.017	0.011	0.002	1.200	0.033

Supplementary Table 2: Site 2 mid slope lower, Wadi Maqam. Heights refer to Supplementary Fig. 2. Fe data in wt%.

Height (m)	Locality	Number	Lith.	Fe_T	Fe_{Carb}	Fe_{Ox}	Fe_{Mag}	Fe_{Py}	Fe_{HR}/Fe_T	Fe_{Py}/Fe_{HR}	Fe/Al
0	OMO13	46	Carbonate	0.090	0.019	0.053	0.005	<0.001	0.880	0.000	
1	OMO13	47	Carbonate	0.060	0.014	0.030	0.008	<0.001	0.900	0.000	
2	OMO13	48	Carbonate	0.060	0.009	0.030	0.003	<0.001	0.690	0.000	
3.4	OMO13	51	Carbonate	0.420	0.030	0.052	0.007	0.001	0.710	0.007	
3.7	OMO13	50	Carbonate	2.040	0.009	1.539	0.018	0.000	0.770	0.000	3.56
4	OMO13	49	Carbonate	0.110	0.020	0.262	0.013	0.002	0.780	0.011	
5	OMO13	52	Carbonate	0.150	0.009	0.102	0.008	0.000	0.710	0.000	
6	OMO13	53	Carbonate	0.120	0.016	0.066	0.007	0.003	0.760	0.033	
9	OMO13	54	Carbonate	0.160	0.008	0.117	0.006	0.002	0.820	0.015	
13	OMO13	55	Carbonate	0.080	0.005	0.056	0.005	0.001	0.840	0.015	
17	OMO13	56	Carbonate	0.150	0.040	0.077	0.007	0.001	0.860	0.008	
21	OMO13	57	Carbonate	0.050	0.000	0.035	0.006	0.001	0.830	0.024	
25	OMO13	58	Carbonate	0.130	0.000	0.096	0.009	0.001	0.790	0.010	
29	OMO13	59	Carbonate	0.130	0.002	0.090	0.009	0.001	0.790	0.010	
33	OMO13	60	Carbonate	0.100	0.000	0.074	0.004	0.001	0.800	0.013	
37	OMO13	61	Carbonate	0.100	0.000	0.065	0.007	0.001	0.730	0.014	
42	OMO13	62	Carbonate	0.170	0.005	0.108	0.004	0.001	0.710	0.008	
45	OMO13	63	Carbonate	0.120	0.006	0.074	0.005	0.001	0.690	0.012	
49	OMO13	64	Carbonate	0.090	0.027	0.038	0.004	0.001	0.810	0.014	
53	OMO13	65	Carbonate	0.100	0.010	0.060	0.006	<0.001	0.740	0.000	
55.2	Maqam	A8	Carbonate	0.280	0.067	0.133	0.014	<0.001	0.770	0.000	
56.1	OMO	83	Carbonate	0.160	0.023	0.081	0.015	0.001	0.750	0.008	
57.3	Maqam	A7	Carbonate	0.270	0.071	0.139	0.020	0.002	0.860	0.009	
58	MAQ1	1	Siliciclastic	0.646	0.023	0.433	0.009	0.002	0.722	0.004	0.444
59.3	MAQ1	2	Siliciclastic	0.751	0.022	0.473	0.011	0.003	0.677	0.006	0.426
60.6	MAQ1	3	Siliciclastic	0.895	0.097	0.540	0.035	0.003	0.754	0.004	0.845
62	MAQ1	4	Siliciclastic	1.348	0.075	0.880	0.012	0.002	0.719	0.002	0.648

62.3	MAQ1	5	Siliciclastic	3.507	0.088	2.162	0.060	0.001	0.659	0.000	0.461
62.36	MAQ1	6	Siliciclastic	3.865	0.129	2.844	0.130	<0.001	0.803	0.000	0.788
62.42	MAQ1	7	Siliciclastic	3.745	0.105	2.646	0.052	<0.001	0.749	0.000	0.603
62.48	MAQ1	8	Siliciclastic	3.178	1.242	1.567	0.047	<0.001	0.899	0.000	0.971
62.53	MAQ1	9	Siliciclastic	2.692	1.462	0.961	0.030	<0.001	0.911	0.000	1.482
62.59	MAQ1	10	Siliciclastic	2.391	1.406	0.926	0.012	<0.001	0.980	0.000	1.551
62.65	MAQ1	11	Siliciclastic	2.815	1.229	1.154	0.036	<0.001	0.859	0.000	1.201
62.71	MAQ1	12	Siliciclastic	2.693	1.381	0.925	0.041	0.001	0.872	0.000	1.187
62.77	MAQ1	13	Siliciclastic	2.626	0.641	1.580	0.045	0.000	0.863	0.000	0.666
62.83	MAQ1	14	Siliciclastic	2.179	1.121	0.759	0.023	0.001	0.873	0.001	0.995
62.88	MAQ1	15	Siliciclastic	2.039	0.906	0.792	0.021	0.001	0.843	0.001	0.647
62.94	MAQ1	16	Siliciclastic	1.979	1.017	0.749	0.031	0.002	0.909	0.001	1.193
63	MAQ1	17	Siliciclastic	2.694	0.409	1.635	0.071	0.001	0.786	0.000	1.199
63.2	MAQ1	18	Siliciclastic	0.966	0.353	0.387	0.006	0.001	0.773	0.001	0.424
63.4	MAQ1	19	Siliciclastic	0.951	0.336	0.499	0.015	0.002	0.896	0.002	0.497
63.8	MAQ1	20	Siliciclastic	1.693	0.327	1.019	0.025	<0.001	0.810	0.000	0.683
64	MAQ1	21	Siliciclastic	1.990	0.248	1.306	0.042	0.001	0.803	0.001	0.664
64.1	MAQ1	22	Siliciclastic	2.089	0.576	1.096	0.027	<0.001	0.813	0.000	0.692
64.2	MAQ1	23	Siliciclastic	2.353	0.578	1.180	0.046	<0.001	0.767	0.000	0.809
64.3	MAQ1	24	Siliciclastic	1.546	0.572	0.719	0.019	<0.001	0.848	0.000	0.737
64.6	MAQ1	25	Carbonate	1.639	0.403	0.845	0.031	0.002	0.782	0.002	0.720
64.8	MAQ1	26	Carbonate	1.630	0.355	0.866	0.036	0.001	0.772	0.001	0.616
65	MAQ1	27	Carbonate	1.729	0.422	0.861	0.038	0.001	0.765	0.001	0.675
65.2	MAQ1	28	Carbonate	2.451	0.381	1.324	0.030	0.001	0.708	0.001	0.631
65.6	MAQ1	29	Carbonate	2.195	0.196	1.374	0.079	0.004	0.753	0.002	0.708
65.9	MAQ1	30	Carbonate	0.562	0.447	0.037	0.000	0.007	0.873	0.014	7.622
66.2	MAQ1	31	Carbonate	0.393	0.079	0.264	0.001	0.001	0.881	0.003	
67.5	MAQ1	32	Carbonate	0.716	0.411	0.208	0.000	0.003	0.869	0.005	1.346
68	MAQ1	33	Carbonate	0.792	0.126	0.465	0.003	0.003	0.753	0.005	0.505
68.2	MAQ1	34	Carbonate	0.308	0.084	0.149	0.000	<0.001	0.756	0.000	0.564
68.7	MAQ1	35	Carbonate	1.213	0.239	0.646	0.020	0.003	0.748	0.003	0.516
74.1	Maqam	273	Carbonate	0.110	0.001	0.083	0.015	<0.001	0.900	0.000	

76	Maqam	277	Carbonate	0.110	0.003	0.065	0.015	0.001	0.740	0.012	
76.7	Maqam	278B	Carbonate	0.110	0.005	0.058	0.016	<0.001	0.720	0.000	
78.4	Maqam	281	Carbonate	0.060	0.002	0.025	0.011	0.001	0.700	0.026	
81	Maqam	287	Carbonate	0.120	0.068	0.028	0.017	<0.001	0.930	0.000	
86	OM08	30	Carbonate	0.420	0.033	0.265	0.019	0.001	0.770	0.003	
88	OM08	33	Carbonate	0.620	0.053	0.322	0.020	0.014	0.660	0.034	0.540
90	OM08	38	Carbonate	0.390	0.054	0.203	0.019	0.015	0.740	0.052	
96	OM10	211	Carbonate	0.220	0.019	0.107	0.019	0.001	0.660	0.007	
98	OM10	216	Carbonate	0.320	0.016	0.187	0.019	0.001	0.700	0.004	
102	OM10	223	Carbonate	1.280	0.044	0.557	0.026	0.001	0.490	0.002	1.429
109	OMO	107	Carbonate	0.370	0.131	0.058	0.019	0.001	0.710	0.002	
110	OM10	237	Carbonate	0.310	0.117	0.124	0.019	<0.001	0.670	0.005	

Supplementary Table 3: Site 3 mid slope upper, Wadi Shuyab. Heights refer to Supplementary Fig. 3. Fe data in wt%.

Height (m)	Locality	Number	Lith.	Fe_T	Fe_{Carb}	Fe_{Ox}	Fe_{Mag}	Fe_{Py}	Fe_{HR}/Fe_T	Fe_{Py}/Fe_{HR}	Fe/Al
-650	OMO	113	Carbonate	0.330	0.128	0.055	0.021	0.001	0.620	0.000	
-550	OMO	128	Carbonate	0.610	0.309	0.081	0.024	0.001	0.690	0.000	0.686
-450	OMO	138	Carbonate	0.560	0.265	0.062	0.026	0.001	0.630	0.000	0.688
-350	OMO	148	Carbonate	0.560	0.226	0.069	0.024	0.001	0.570	0.000	0.589
-250	OMO	161	Carbonate	0.360	0.083	0.100	0.021	<0.001	0.570	0.000	
-150	OMO	168	Carbonate	0.690	0.339	0.104	0.030	<0.001	0.690	0.000	0.807
-50	OMO	172A	Carbonate	0.190	0.088	0.064	0.015	<0.001	0.890	0.000	
10	OMO	177	Carbonate	0.320	0.169	0.062	0.019	<0.001	0.780	0.000	
100	OMO	182	Carbonate	0.150	0.078	0.040	0.016	<0.001	0.890	0.000	
200	OMO	49B	Carbonate	0.420	0.185	0.081	0.017	0.001	0.670	0.000	
222	SHUY2	7	Siliciclastic	3.767	0.138	0.692	0.113	0.004	0.252	0.004	0.458
226.2	SHUY2	6	Siliciclastic	3.824	0.126	0.803	0.106	0.001	0.271	0.001	0.461
227.9	SHUY2	5	Siliciclastic	3.547	0.048	0.828	0.061	0.004	0.265	0.004	0.422
234.05	SHUY1	12	Siliciclastic	2.118	0.081	0.832	0.069	0.001	0.464	0.001	0.491
234.2	SHUY1	13	Carbonate	2.495	0.344	1.437	0.054	0.006	0.738	0.003	1.755
234.3	SHUY1	14	Carbonate	2.184	1.412	0.358	0.008	0.005	0.817	0.003	1.612
234.55	SHUY1	15	Carbonate	2.383	0.327	1.454	0.048	0.002	0.768	0.001	1.894
234.75	SHUY1	17	Carbonate	0.448	0.103	0.125	0.003	0.002	0.517	0.008	
234.85	SHUY1	18	Siliciclastic	0.702	0.113	0.436	0.000	0.002	0.786	0.004	4.118
235	SHUY1	19	Carbonate	0.697	0.158	0.333	0.000	0.005	0.710	0.010	0.984
235.15	SHUY1	21	Carbonate	1.016	0.538	0.334	0.000	0.005	0.863	0.006	1.954
235.15	SHUY1	20	Carbonate	1.574	0.937	0.356	0.002	0.005	0.826	0.004	2.336
235.65	SHUY1	22	Carbonate	1.330	0.888	0.208	0.000	0.004	0.827	0.004	1.591
235.95	SHUY1	23	Carbonate	1.415	0.871	0.267	0.002	0.006	0.809	0.005	1.737
236.2	SHUY1	24	Carbonate	0.473	0.083	0.248	0.000	0.003	0.705	0.008	
236.3	SHUY1	25	Carbonate	0.643	0.126	0.355	0.000	0.002	0.752	0.005	1.461
236.5	SHUY1	26	Carbonate	1.819	1.141	0.495	0.016	0.005	0.911	0.003	4.068
236.75	SHUY1	27	Carbonate	0.487	0.190	0.164	0.003	0.003	0.739	0.008	
237.1	SHUY1	28	Siliciclastic	2.984	0.064	1.254	0.098	0.001	0.474	0.001	0.735

237.4	SHUY1	29	Siliciclastic	0.454	0.225	0.091	0.000	0.002	0.701	0.007	
237.5	SHUY1	29A	Siliciclastic	2.938	0.073	0.556	0.059	0.001	0.235	0.002	0.414
237.8	SHUY1	30	Siliciclastic	0.897	0.279	0.134	0.016	0.002	0.480	0.004	0.997
238.5	SHUY1	32	Carbonate	4.132	0.043	0.892	0.229	<0.001	0.282	0.000	0.451
239	SHUY2	2	Siliciclastic	4.988	0.012	1.705	0.275	<0.001	0.400	0.000	0.493
239.1	SHUY1	33	Carbonate	0.454	0.091	0.154	0.006	0.002	0.557	0.008	0.561
239.7	SHUY1	34	Carbonate	4.478	0.075	0.730	0.245	<0.001	0.235	0.000	0.518
240	SHUY2	1	Siliciclastic	5.492	0.046	1.516	0.132	<0.001	0.308	0.000	0.476
241	SHUY1	1	Siliciclastic	2.615	0.120	0.681	0.127	0.005	0.357	0.005	0.430
241.9	SHUY1	2	Siliciclastic	4.405	0.081	1.325	0.385	<0.001	0.407	0.000	0.426
242	SHUY1	3	Carbonate	2.428	0.103	1.474	0.055	<0.001	0.672	0.000	1.339
242.1	SHUY1	4	Carbonate	2.702	0.132	1.942	0.064	0.004	0.793	0.002	1.984
242.3	SHUY1	5	Siliciclastic	1.183	0.026	0.695	0.026	<0.001	0.632	0.000	0.642
242.5	SHUY1	6	Carbonate	2.818	0.082	1.977	0.079	<0.001	0.758	0.000	1.576
242.85	SHUY1	7	Carbonate	2.582	0.048	2.146	0.040	<0.001	0.865	0.000	1.453
243.15	SHUY1	8	Carbonate	2.773	0.068	1.896	0.038	<0.001	0.722	0.000	1.345
243.35	SHUY1	9	Siliciclastic	2.750	0.043	1.751	0.054	0.001	0.673	0.001	1.164
243.75	SHUY1	10	Carbonate	0.377	0.156	0.120	0.000	0.003	0.739	0.010	
243.9	SHUY1	11	Carbonate	0.396	0.124	0.071	0.004	0.002	0.508	0.010	
248.8	SHUY1	34A	Siliciclastic	0.798	0.166	0.191	0.023	<0.001	0.476	0.000	1.250
258.8	SHUY1	34B	Siliciclastic	4.164	0.046	0.740	0.202	<0.001	0.238	0.000	0.461
268.8	SHUY1	35	Siliciclastic	4.199	0.046	0.742	0.189	<0.001	0.233	0.000	0.483
278.8	SHUY1	37	Siliciclastic	3.941	0.074	0.797	0.302	<0.001	0.298	0.000	0.516
288.8	SHUY1	38	Siliciclastic	4.480	0.064	1.044	0.308	<0.001	0.316	0.000	0.542
298.8	SHUY1	40	Siliciclastic	4.675	0.050	0.830	0.215	<0.001	0.234	0.000	0.536
303.8	SHUY1	41	Siliciclastic	3.904	0.086	1.630	0.157	<0.001	0.480	0.000	0.891
308.8	SHUY1	42	Siliciclastic	4.582	0.033	1.075	0.240	<0.001	0.294	0.000	0.501
318.8	SHUY1	43	Siliciclastic	4.641	0.052	1.130	0.334	<0.001	0.327	0.000	0.547
328.8	SHUY1	44	Siliciclastic	4.846	0.041	1.290	0.265	<0.001	0.329	0.000	0.547
338.8	SHUY1	45	Siliciclastic	4.251	0.043	1.033	0.288	<0.001	0.321	0.000	0.485

Supplementary Table 4: Site 4 distal slope, Wadi Wasit South. Heights refer to Supplementary Fig. 4. Fe data in wt%.

Height (m)	Locality	Number	Lith.	Fe_T	Fe_{Carb}	Fe_{Ox}	Fe_{Mag}	Fe_{Py}	Fe_{HR}/Fe_T	Fe_{Py}/Fe_{HR}	Fe/Al	$\delta^{34}S_{py}$ (‰)
0	WWS1	84	Carbonate	3.290	0.628	2.115	0.018	0.002	0.840	0.001	5.463	
0.6	WWS1	83	Siliciclastic	2.010	0.401	1.346	0.052	0.003	0.890	0.001	0.920	
1.2	WWS1	82	Siliciclastic	2.410	0.383	1.611	0.020	0.001	0.840	0.001	1.085	4.3
1.8	WWS1	81	Siliciclastic	1.680	0.007	1.415	0.129	0.004	0.930	0.002	0.233	
2.1	WWS1	80	Siliciclastic	1.850	0.011	1.678	0.146	0.003	0.990	0.001	0.271	
2.8	WWS1	79	Siliciclastic	3.450	0.161	2.917	0.033	0.002	0.900	0.000	1.215	
3.5	WWS1	78	Carbonate	0.250	0.086	0.094	-0.003	0.002	0.710	0.012		
3.7	WWS1	77	Siliciclastic	3.480	0.081	2.646	0.086	0.004	0.810	0.001	0.726	
4	WWS1	76	Siliciclastic	3.650	0.071	3.049	0.069	0.002	0.880	0.001	0.939	
4.5	WWS1	75	Siliciclastic	3.050	0.017	2.503	0.076	0.002	0.850	0.001	0.854	
5	WWS1	74	Siliciclastic	2.120	0.016	1.821	0.083	0.003	0.910	0.002	0.379	
5.8	WWS1	72	Carbonate	4.250	0.122	3.157	0.050	0.002	0.780	0.001	2.593	
6.2	WWS1	71	Carbonate	1.840	0.159	1.278	0.032	<0.001	0.800	0.000	0.655	
7	WWS1	70	Carbonate	1.430	0.106	0.882	0.052	0.001	0.730	0.001	0.443	
7.7	WWS1	68	Siliciclastic	1.370	0.097	0.877	0.042	0.001	0.740	0.001	0.573	
10.3	WWS1	67	Carbonate	0.810	0.152	0.443	0.028	0.001	0.770	0.002	1.170	
11	WWS1	66	Siliciclastic	1.200	0.106	0.933	0.051	<0.001	0.910	0.000	0.544	
12	WWS1	65	Carbonate	0.260	0.188	0.049	0.013	<0.001	0.950	0.001		24.5
13	WWS1	64	Carbonate	0.900	0.109	0.571	0.034	<0.001	0.790	0.000	0.434	
14	WWS1	63	Carbonate	0.220	0.043	0.103	0.017	<0.001	0.730	0.001		
16	WWS1	62	Carbonate	1.070	0.185	0.634	0.032	0.002	0.800	0.002	0.752	
17.2	WWS1	61	Carbonate	0.480	0.160	0.234	0.019	0.002	0.870	0.004		
18	WWS1	60	Carbonate	0.990	0.233	0.483	0.024	0.003	0.750	0.004	1.005	
19	WWS1	59	Siliciclastic	2.930	0.096	1.952	0.047	0.001	0.720	0.001	1.327	14
19.5	WWS1	58	Carbonate	1.580	0.315	0.783	0.030	0.003	0.720	0.002	0.807	
20	WWS1	57	Siliciclastic	0.880	0.293	0.381	0.024	0.003	0.800	0.004	0.779	
20.8	WWS1	56	Carbonate	0.590	0.098	0.315	0.021	0.001	0.740	0.002	0.491	17.6
21.2	WWS1	55	Siliciclastic	1.980	0.174	1.323	0.059	0.004	0.790	0.002	1.177	

21.9	WWS1	54	Carbonate	0.480	0.073	0.287	0.018	0.002	0.790	0.004		
22.2	WWS1	52	Carbonate	0.280	0.230	0.152	0.014	0.001	1.400	0.001		
23	WWS1	53	Siliciclastic	1.330	0.185	1.524	0.037	0.001	1.310	0.001	0.751	
23.9	WWS1	51	Siliciclastic	0.660	0.065	0.403	0.022	0.002	0.740	0.003	0.842	17.1
24.9	WWS1	50	Siliciclastic	2.210	0.188	1.753	0.035	0.005	0.900	0.002	1.039	14.2
25.4	WWS1	49	Siliciclastic	2.450	0.044	1.466	0.036	0.003	0.630	0.002	0.560	
26.8	WWS1	48	Carbonate	0.730	0.259	0.270	0.020	0.001	0.750	0.002	0.664	
27.8	WWS1	47	Carbonate	0.900	0.351	0.312	0.022	<0.001	0.760	0.001	0.910	
29.2	WWS1	46	Carbonate	0.550	0.145	0.247	0.019	0.001	0.740	0.002	0.790	
30.3	WWS1	45	Carbonate	0.310	0.129	0.110	0.020	0.002	0.840	0.008		
32.3	WWS1	44	Carbonate	0.690	0.027	0.024	0.002	0.002	0.080	0.033	0.824	
34.2	WWS1	43	Carbonate	0.250	0.125	0.076	0.018	0.002	0.890	0.009		
36	WWS1	42	Carbonate	2.250	0.240	1.710	0.033	0.001	0.880	0.001	1.089	
37	WWS1	41	Carbonate	0.260	0.096	0.093	0.017	<0.001	0.790	0.000		
39	WWS1	40	Carbonate	0.330	0.121	0.108	0.023	<0.001	0.770	0.000		
41	WWS1	39	Siliciclastic	1.370	0.135	0.707	0.038	<0.001	0.640	0.000	0.718	
42	WWS1	38	Carbonate	1.750	0.165	1.256	0.044	<0.001	0.840	0.000	1.006	
43	WWS1	37	Carbonate	0.700	0.118	0.306	0.020	<0.001	0.630	0.001	0.427	
45	WWS1	36	Carbonate	0.480	0.000	0.000	0.000	0.001	0.000	0.950		
46.2	WWS1	35	Carbonate	0.280	0.127	0.090	0.014	<0.001	0.820	0.001		
47	WWS1	34	Siliciclastic	2.050	0.095	1.550	0.057	0.001	0.830	0.000	1.006	
47.5	WWS1	33	Siliciclastic	2.240	0.086	1.259	0.074	0.001	0.640	0.000	1.268	
48	WWS1	32	Carbonate	0.420	0.237	0.100	0.006	0.001	0.820	0.002		
49	WWS1	30	Carbonate	0.590	0.301	0.083	0.010	0.034	0.730	0.079	1.419	
51	WWS1	29	Carbonate	0.510	0.108	0.203	0.008	0.001	0.630	0.002	0.482	
53	WWS1	28	Carbonate	1.110	0.342	0.520	0.011	<0.001	0.780	0.001	1.803	
57	WWS1	26	Carbonate	0.300	0.161	0.062	0.004	0.001	0.770	0.005		
60.8	WWS1	25	Carbonate	0.350	0.219	0.055	0.005	0.001	0.810	0.004		
63.8	WWS1	22	Carbonate	0.490	0.253	0.082	0.010	0.001	0.710	0.003		
69.8	WWS1	20	Carbonate	0.410	0.156	0.081	0.006	0.001	0.600	0.004		
75	WWS1	17	Carbonate	0.470	0.270	0.093	0.007	0.001	0.780	0.002		
80.8	WWS1	16	Carbonate	0.930	0.419	0.113	0.020	0.002	0.600	0.003	0.829	

83.7	WWS1	15	Carbonate	0.690	0.410	0.149	0.010	0.002	0.830	0.003	1.193
87	WWS1	13	Carbonate	0.920	0.398	0.198	0.017	0.001	0.670	0.001	1.172
91	WWS1	11	Carbonate	0.470	0.255	0.132	0.007	0.001	0.830	0.002	
93	WWS1	10	Carbonate	0.480	0.256	0.143	0.006	0.001	0.850	0.002	
95	WWS1	8	Carbonate	0.480	0.272	0.152	0.006	<0.001	0.900	0.000	
97	WWS1	6	Carbonate	0.370	0.153	0.155	0.009	<0.001	0.860	0.001	
101.2	WWS1	3	Carbonate	0.860	0.219	0.480	0.009	0.001	0.830	0.001	0.726
104.2	WWS1	1	Carbonate	0.980	0.158	0.501	0.013	0.001	0.690	0.002	0.631

Supplementary Table 5: Site 4 distal slope upper, Radio Tower. Heights refer to Supplementary Fig. 5. Fe data in wt%.

Height (m)	Locality	Number	Lithology	$\delta^{13}C$ (‰PBD)	$\delta^{18}O$ (‰PBD)	Fe_T	Fe_{Carb}	Fe_{Ox}	Fe_{Mag}	Fe_{Py}	Fe_{HR}/Fe_T	Fe_{Py}/Fe_{HR}	Fe/Al
0	RT1	1	Carbonate	-1.803	-4.238	0.380	0.082	0.189	0.002	<0.001	0.720	0.001	
1	RT1	2	Carbonate	-1.561	-4.710	0.640	0.095	0.278	0.005	0.001	0.590	0.002	0.751
2	RT1	3	Carbonate	-1.522	-4.614	0.780	0.142	0.317	0.001	<0.001	0.590	0.000	1.186
3	RT1	4	Carbonate	-1.183	-5.183	0.440	0.206	0.096	0.000	<0.001	0.680	0.001	
4	RT1	5	Carbonate	-1.861	-4.593	1.030	0.135	0.509	0.006	<0.001	0.630	0.000	1.001
4.5	RT1	4A	Siliciclastic			1.260	0.215	0.648	0.021	0.001	0.700	0.001	0.946
5.2	RT1	6	Carbonate	-0.859	-3.918	0.760	0.062	0.337	0.004	0.001	0.530	0.002	0.632
6.2	RT1	7	Carbonate	-0.408	-4.327	0.490	0.252	0.096	0.000	<0.001	0.700	0.001	
7.4	RT1	8	Carbonate	-1.039	-4.179	0.670	0.048	0.326	0.007	0.001	0.570	0.002	0.535
8.7	RT1	9	Carbonate	-0.828	-4.531	0.490	0.049	0.189	0.000	<0.001	0.490	0.002	
9.5	RT1	10	Carbonate	-0.471	-4.168	0.480	0.210	0.209	0.000	0.001	0.860	0.001	
10.3	RT1	11	Carbonate	-0.414	-4.228	0.680	0.046	0.322	0.003	0.001	0.550	0.002	0.628
11.2	RT1	12	Carbonate	-0.463	-3.510	0.500	0.036	0.193	0.003	<0.001	0.460	0.002	0.637
12.3	RT1	13	Carbonate	-0.876	-3.020	0.730	0.034	0.375	0.007	<0.001	0.570	0.000	0.693
13.1	RT1	14	Carbonate	0.292	-3.365	0.340	0.201	0.084	0.000	<0.001	0.830	0.001	
14.1	RT1	15	Carbonate	0.535	-3.898	0.390	0.119	0.093	0.000	<0.001	0.540	0.000	
15.1	RT1	16	Carbonate	-0.012	-3.150	0.740	0.021	0.297	0.007	<0.001	0.440	0.000	0.487
15.6	RT1	17	Carbonate	0.320	-2.142	0.630	0.019	0.196	0.003	<0.001	0.340	0.001	0.516
16.5	RT1	18	Carbonate	0.694	-4.931	0.240	0.075	0.060	0.000	0.001	0.550	0.005	
17.5	RT1	19	Carbonate	1.087	-4.202	0.440	0.115	0.129	0.001	<0.001	0.560	0.001	
18.1	RT1	20	Carbonate	0.698	-4.299	0.480	0.031	0.169	0.001	0.001	0.420	0.003	
19	RT1	21	Carbonate	0.989	-4.057	0.640	0.037	0.284	0.001	<0.001	0.500	0.001	0.687
19.5	RT1	22	Carbonate			1.150	0.085	0.699	0.021	<0.001	0.700	0.000	1.778
20.6	RT1	23	Carbonate	3.096	-2.556	0.530	0.032	0.445	0.006	<0.001	0.920	0.000	1.287
21.6	RT1	24	Carbonate	4.778	-3.242	0.420	0.025	0.138	0.000	<0.001	0.380	0.002	
22	RT1	25	Carbonate	4.870	-2.981	0.420	0.024	0.138	0.000	0.001	0.390	0.003	

23.5	RT1	26	Carbonate	6.733	-2.812	0.370	0.046	0.119	0.000	<0.001	0.430	0.002	
24.1	RT1	27	Carbonate	6.209	-4.821	0.310	0.099	0.101	0.000	<0.001	0.640	0.001	
25	RT1	28	Carbonate	2.811	-3.868	0.450	0.188	0.120	0.001	0.001	0.680	0.002	
27	RT1	30	Carbonate	3.576	-3.500	0.270	0.061	0.083	0.000	<0.001	0.520	0.003	
27.5	RT1	31	Carbonate	-1.676	-5.087	3.110	0.003	1.682	0.035	0.001	0.550	0.000	1.137
27.7	RT1	32	Carbonate	-0.885	-2.382	2.110	0.023	1.308	0.023	<0.001	0.640	0.000	1.328
28	RT1	33	Carbonate	-0.476	-2.294	2.490	0.003	1.661	0.043	0.001	0.690	0.000	1.266
28.1	RT1	34	Carbonate	1.330	-4.297	0.370	0.166	0.086	0.003	<0.001	0.690	0.000	
28.8	RT1	35	Carbonate	-0.458	-4.622	1.850	0.161	1.139	0.059	<0.001	0.740	0.000	1.545
29	RT1	36	Carbonate	1.657	-4.193	0.300	0.243	0.011	0.000	<0.001	0.860	0.000	
30.9	RT1	37	Carbonate	-1.667	-2.329	1.520	0.059	1.208	0.024	<0.001	0.850	0.000	1.002
34.2	RT1	38	Carbonate	-0.766	-4.449	0.320	0.105	0.101	0.003	<0.001	0.650	0.000	
35.5	RT1	39	Carbonate	0.011	-5.402	0.860	0.238	0.366	0.012	<0.001	0.720	0.000	1.998
36.1	RT1	40	Carbonate	0.387	-4.877	0.430	0.222	0.110	0.002	<0.001	0.770	0.000	
37.9	RT1	41	Carbonate	-0.474	-4.151	1.480	0.084	0.063	0.005	<0.001	0.100	0.002	1.789
39	RT1	42	Carbonate	0.182	-4.411	0.280	0.171	0.529	0.01	0.001	2.550	0.002	

Supplementary Table 6: Site 5 seamount, Baid. Heights refer to Supplementary Fig. 6. Fe data in wt%.

Height (m)	Locality	Number	Lithology	Fe_T	Fe_{Carb}	Fe_{Ox}	Fe_{Mag}	Fe_{Py}	Fe_{HR}/Fe_T	Fe_{Py}/Fe_{HR}
0	BAID	1	Carbonate	0.375	0.242	0.016	0.013	<0.001	0.721	0.000
1	BAID	2	Carbonate	0.465	0.205	0.043	0.010	<0.001	0.557	0.000
2	BAID	3	Carbonate	0.252	0.128	0.021	0.010	<0.001	0.630	0.000
3	BAID	4	Carbonate	0.397	0.000	0.138	0.016	<0.001	0.387	0.000
3.2	BAID	5	Carbonate	0.000	0.000	0.000	0.001	<0.001		0.000
3.7	BAID	6	Carbonate	0.157	0.003	0.045	0.015	<0.001	0.400	0.000
4.5	BAID	7	Carbonate	0.334	0.000	0.109	0.015	<0.001	0.370	0.000
6	BAID	8	Carbonate	0.040	0.000	0.011	0.000	<0.001	0.260	0.000
6.3	BAID	9	Carbonate	0.000	0.000	0.000	0.000	<0.001		0.000
7.3	BAID	10	Carbonate	0.229	0.000	0.085	0.000	<0.001	0.373	0.000
7.8	BAID	11	Carbonate	0.190	0.000	0.001	0.000	<0.001	0.004	0.000
8	BAID	12	Carbonate	0.179	0.000	0.068	0.000	<0.001	0.379	0.000
11.5	BAID	13	Carbonate	0.000	0.000	0.000	0.000	<0.001		0.000
12	BAID	14	Carbonate	0.011	0.000	0.000	0.000	<0.001	0.099	0.000
12.5	BAID	15	Carbonate	0.212	0.005	0.075	0.000	<0.001	0.378	0.000
13	BAID	16	Carbonate	0.199	0.004	0.075	0.000	<0.001	0.402	0.000
13.2	BAID	17	Carbonate	0.021	0.000	0.011	0.000	<0.001	0.644	0.000
13.3	BAID	18	Carbonate	0.091	0.000	0.057	0.000	<0.001	0.627	0.000

Supplementary Table 7: Site 6 Deep Basin, Buday'ah. Heights refer to Supplementary Fig. 7. Fe data in wt%.

Height (m)	Locality	Number	Lithology	Fe_T	Fe_{Carb}	Fe_{Ox}	Fe_{Mag}	Fe_{Py}	Fe_{HR}/Fe_T	Fe_{Py}/Fe_{HR}	Fe/Al	$\delta^{34}S_{py}$ (‰)
0.1	BUD1	1	Radiolarian	4.346	0.147	0.981	0.236	<0.001	0.314	0.000	1.799	
0.5	BUD1	2	Radiolarian	5.876	0.077	2.914	0.287	<0.001	0.543	0.000	1.368	
0.7	BUD1	3	Radiolarian	3.129	0.054	1.763	0.119	<0.001	0.618	0.000	0.892	
1.1	BUD1	4	Radiolarian	0.961	0.000	0.585	0.013	<0.001	0.622	0.000	0.249	
1.3	BUD1	5	Radiolarian	1.514	0.000	0.969	0.029	<0.001	0.660	0.000	0.292	
1.8	BUD1	6	Radiolarian	1.107	0.079	0.563	0.015	<0.001	0.593	0.000	0.979	
2.4	BUD1	7	Radiolarian	2.432	0.000	1.498	0.021	<0.001	0.625	0.000	0.352	
2.9	BUD1	8	Radiolarian	3.506	0.010	2.814	0.056	0.002	0.822	0.001	0.500	
3.5	BUD1	9	Radiolarian	4.627	0.044	2.637	0.224	<0.001	0.628	0.000	0.794	3.2
3.6	BUD1	10	Radiolarian	3.338	0.005	2.487	0.067	<0.001	0.766	0.000	0.490	7.7
4.8	BUD1	11	Siliciclastic	5.396	0.002	4.606	0.121	0.003	0.877	0.001	0.808	
5.0	BUD1	12	Siliciclastic	4.319	0.057	1.993	0.219	<0.001	0.525	0.000	0.585	
5.3	BUD1	13	Siliciclastic	21.226	0.035	15.461	0.756	<0.001	0.766	0.000	4.008	
5.7	BUD1	14	Siliciclastic	2.236	0.030	0.327	0.063	<0.001	0.188	0.000	0.353	7.7
6.1	BUD1	15	Siliciclastic	3.871	0.030	2.065	0.142	0.014	0.581	0.006	0.727	-15
6.3	BUD1	16	Siliciclastic	2.484	0.011	1.577	0.038	0.003	0.656	0.002	0.343	2.2
6.4	BUD1	17	Siliciclastic	5.048	0.036	2.900	0.173	0.019	0.620	0.006	0.779	
6.6	BUD1	18	Siliciclastic	3.975	0.047	1.240	0.209	<0.001	0.376	0.000	0.613	
7.3	BUD1	19	Siliciclastic	3.503	0.030	1.478	0.126	<0.001	0.467	0.000	0.537	
7.4	BUD1	20	Siliciclastic	4.291	0.123	1.308	0.235	0.001	0.388	0.000	0.710	
7.6	BUD1	21	Siliciclastic	4.239	0.068	1.205	0.222	<0.001	0.353	0.000	0.702	
7.9	BUD1	22	Siliciclastic	6.842	0.149	1.648	0.414	<0.001	0.323	0.000	1.107	
8.0	BUD1	23	Carbonate	2.258	0.337	0.330	0.108	<0.001	0.343	0.000	0.811	14.2
8.3	BUD1	24	Siliciclastic	7.625	0.184	1.923	0.536	0.001	0.347	0.000	1.178	
8.6	BUD1	25	Siliciclastic	5.940	0.087	1.540	0.416	<0.001	0.344	0.000	0.966	
9.1	BUD1	26	Siliciclastic	4.553	0.216	0.995	0.243	0.001	0.319	0.001	0.869	
9.5	BUD1	27	Siliciclastic	3.986	0.035	1.289	0.129	0.003	0.365	0.002	0.581	

9.9	BUD1	28	Carbonate	0.703	0.206	0.156	0.024	0.001	0.551	0.003	0.705	10.5
10.3	BUD1	29	Siliciclastic	1.304	0.144	0.234	0.036	0.002	0.319	0.004	0.503	27.6
10.9	BUD1	31	Siliciclastic	1.098	0.185	0.193	0.022	0.001	0.366	0.003	0.603	17.4
11.0	BUD1	32	Siliciclastic	2.022	0.179	0.334	0.077	0.003	0.293	0.004	0.650	
11.6	BUD1	33	Siliciclastic	3.773	0.049	1.061	0.124	0.001	0.327	0.001	0.519	
12.2	BUD1	34	Carbonate	0.746	0.220	0.110	0.030	<0.001	0.483	0.000	0.938	16.4
13.0	BUD1	35	Siliciclastic	0.596	0.169	0.106	0.024	0.001	0.503	0.002	0.531	
13.1	BUD1	36	Carbonate	0.602	0.289	0.048	0.018	<0.001	0.589	0.000	0.927	
13.3	BUD1	37	Siliciclastic	2.395	0.237	0.514	0.130	<0.001	0.368	0.000	0.570	16.4
14.1	BUD1	38	Carbonate	2.319	0.401	0.288	0.065	0.002	0.326	0.002	1.004	
14.3	BUD1	39	Siliciclastic	3.990	0.069	0.883	0.092	<0.001	0.261	0.000	0.534	
15.2	BUD1	40	Siliciclastic	5.368	0.179	1.424	0.373	<0.001	0.368	0.000	0.795	11.3
16.2	BUD1	41	Carbonate	0.793	0.268	0.121	0.023	0.001	0.520	0.002	0.929	
17.0	BUD1	42	Siliciclastic	1.139	0.194	0.183	0.041	0.003	0.370	0.007	0.584	
17.2	BUD1	43	Carbonate	0.998	0.267	0.188	0.026	<0.001	0.481	0.000	0.768	16.6
17.5	BUD1	44	Siliciclastic	1.959	0.169	0.374	0.077	0.001	0.318	0.002	0.595	
19.5	BUD1	45	Siliciclastic	0.394	0.147	0.071	0.006	0.001	0.571	0.002		
19.9	BUD1	46	Siliciclastic	0.640	0.157	0.118	0.012	0.001	0.450	0.002	0.557	
20.4	BUD2	1	Carbonate	0.514	0.154	0.170	0.011	0.001	0.655	0.004	0.547	32.5
20.6	BUD2	2	Carbonate	2.514	0.131	0.463	0.095	<0.001	0.274	0.000	0.514	23.1
21.0	BUD2	3	Siliciclastic	0.740	0.138	0.169	0.019	0.003	0.444	0.008	0.429	
22.0	BUD2	4	Carbonate	4.768	0.117	1.450	0.355	<0.001	0.403	0.000	0.658	24.9
22.7	BUD2	5	Siliciclastic	0.503	0.138	0.114	0.016	<0.001	0.535	0.005	0.395	
23.9	BUD2	7	Carbonate	3.219	0.219	0.330	0.119	<0.001	0.207	0.000	0.782	
24.6	BUD2	8	Carbonate	1.090	0.188	0.116	0.035	<0.001	0.311	0.001	0.573	
25.4	BUD2	9	Carbonate	2.104	0.147	0.279	0.051	<0.001	0.226	0.000	0.547	
27.0	BUD2	10	Siliciclastic	0.477	0.170	0.221	0.008	<0.001	0.834	0.000		
28.9	BUD2	11	Siliciclastic	0.678	0.151	0.203	0.017	0.004	0.553	0.010	0.470	
31.5	BUD2	12	Carbonate	2.234	0.219	0.334	0.100	0.001	0.293	0.002	0.607	9.9
33.1	BUD2	13	Carbonate	1.959	0.236	0.211	0.066	0.002	0.263	0.004	0.849	
34.9	BUD2	14	Carbonate	4.675	0.072	1.119	0.191	0.001	0.296	0.001	0.702	

Supplementary Note 1: Section Descriptions

The principle platform site utilized for Fe-speciation is the Saiq Plateau (Supplementary Fig. 1). Facies include pack-grainstone dominated textures representing shoal to fore-shoal deposits[4]. The PTB is characterized by early dolomites and, locally, a poorly developed laminated shale or a breccia level. As such it was not possible to sample the shale directly, however there is no apparent Fe-enrichment from this lithology, as is clear for the slope section in red-orange marls. Bioturbated early marine dolomites then characterize the entire Induan. Olive gray-purple shales and yellow marls characterize the end-Dienerian with a switch to limestones in the lower Smithian. This increase in siliciclastic may reflect the increased weathering rates observed elsewhere at the peak of sedimentation rates in the Early Triassic[5]. Small foraminifera and the bivalve *Claraia* are seen in carbonates at the end-Dienerian.

The Wasit Block (site 2) represents a block of Late-Permian and Griesbachian platform carbonates that were reworked into slope breccias during the Dienerian[2]. Fe-speciation analysis was not performed on this section but it holds important information with respect to biodiversity during the Griesbachian[2,6], and so is included as a site in the discussion of anoxia.

Slope deposits of the Sumeini group (site 3) extend from the middle Permian to the Cretaceous and are separated in two contiguous sections; Wadi Maqam and Wadi Shuyab (Supplementary Figs. 2 and 3). Deposits include cherty dolomudstone of the late Permian, red to yellow marls, silts and shales around the PTB and platy limestones and shales for the Early Triassic[1]. The second section for the middle slope Sumeini group is Wadi Shuyab (Supplementary Fig. 3) that represents a continuation of the Maqam section. Sedimentation rates increased significantly here leading to an expanded Smithian of ~900m and hence lower resolution sampling. Additionally these sections include slump and breccia deposits linked to regional tectonic activity.

Early Triassic distal slope deposits (site 4) come from two sections; Wadi Wasit South (Supplementary Fig. 4) for the Late-Dienerian to mid-Smithian[1] and Radio Tower for the mid-Smithian to Early-Spathian (Supplementary Fig. 5). They represent distal turbidite deposits of platy limestones and shales with no bioturbation.

The Alwa Formation in the Ba'id Exotic (site 5, Supplementary Fig. 6) was deposited on an isolated carbonate platform, situated on a tilted block of the Gondwana margin[7]. Shallow-water carbonate platform deposits occurred from the Wordian to the Wuchiapingian. The block was exposed during the early Induan and resubmerged due to tectonic activity in the late Induan. Subsequently, pelagic ammonoid bearing limestones were deposited. The Alwa Fm. also contains numerous unusual carbonate fabrics, including thrombolites, microbialites, botryoidal calcite (*Frutexites*) and isopachous calcite cements (*Stromatactis*)[8].

Buday'ah (site 6) represents one of the deepest basinal deposits of the Neo-Tethys for the Permian-Triassic period[3]. Formation ages have been refined owing to new bio-stratigraphic correlations from this study, but remain poorly resolved (Supplementary Fig. 7). Red pelagic cephalopod-bearing limestones were deposited onto basement basalt during the Wordian, followed by radiolarian cherts during the Wuchiapingian and mixed carbonate/clastic shales from the Changsingian to the Spathian. Unconformities are present between the Wordian and Wuchiapingian, in the mid-Dienerian, and at the end-Smithian.

Supplementary Note 2: Dilution of Fe_{HR}

In turbidite settings high sedimentation rates can cause a dilution of Fe_{HR}[9,10] and thus decrease of the Fe_{HR}/Fe_T ratio of sediments deposited under an anoxic water column, giving a falsely oxic signal. The mid-slope setting at Sumeini is a turbidite settings, with high sedimentation rate mainly during the mid to late Smithian. Dilution of Fe_{HR} could potentially cause the low Fe_T samples in this section, however they often occur with bioturbation suggesting that the low Fe_T might simply be a consequence of oxic deposition (Supplementary Fig. 1 and 2). The distal-slope settings of the Hawasina group (Wadi Wasit South and Radio Tower) represent distal turbidite environments, which could potentially suffer from such Fe_{HR} dilutions. In the Fe_{HR}/Fe_T data (Supplementary Figs. 4 and 5) subtle trends can be seen at decimeter scale that may reflect such changes in sedimentation rate. The dominance of high Fe_{HR}/Fe_T ratios suggests that sedimentation rates were not high enough to create long periods of falsely oxic signatures.

Supplementary Note 3: Oxidative Weathering

Although we took great care to sample well-preserved rocks, including removing rock surfaces prior to crushing, due to the use of outcrop material here, samples may have potentially been affected by oxidative weathering. However, the majority of shale samples are olive grey with no evidence of Fe oxide staining, suggesting minimal oxidation. The anoxic limestone samples sometimes show a pervasive red to orange coloration that might suggest oxidative weathering. However, such a colouration might also be expected for limestones deposited under ferruginous conditions. We nevertheless consider the possibility for oxidation in more detail. Firstly, it should be noted that pyrite and Fe_{carb} minerals, such as siderite, are the dominant Fe_{HR} minerals that may undergo oxidation. In both cases, however, oxidation will not significantly impact Fe_{HR}/Fe_T or Fe/Al ratios, and hence any interpretation of anoxia remains robust. Oxidation of siderite would transfer Fe_{carb} to the Fe_{ox} pool, and hence this would not affect identification of a ferruginous or euxinic signal. A robust euxinic signature is identified by Fe_{py}/Fe_{HR} ratios >0.7-0.8[10,11] and oxidative weathering has the potential to significantly decrease this ratio by transfering Fe_{py} to Fe_{ox}. If we consider an extreme and highly unlikely scenario,

whereby there was no Fe_{ox} in the primary sample, and all Fe_{py} has been oxidized to Fe_{ox}, with no oxidation of Fe_{carb} to Fe_{ox}, approximately 46% of the anoxic samples would give a euxinic signal. However, Fe_{carb} occurs in 97% of the anoxic samples, indicating that the rocks have not been completely weathered, and hence this extreme scenario is unlikely. The substantial contribution of Fe_{carb} further supports the interpretation of incomplete oxidation as does the fact we were able to analyze $\delta^{34}S_{pyrite}$ in many samples. Therefore more than 50% of the anoxic samples demonstrate unequivocal deposition under ferruginous conditions, and this proportion was likely much higher. While it seems unlikely that a significant proportion of our samples were deposited under euxinic conditions, we cannot rule out the possibility of sporadic euxinia. Nevertheless, our data strongly indicate that anoxia was characterized, mostly, and potentially entirely, by ferruginous conditions.

Supplementary Note 4: Dolomitization

Clarkson et al.[12] explore the role of late stage dolomitization on Fe-speciation data using samples from the Sumeini Early Triassic section (Site 3), where an oblique dolomitization front cross-cuts the outcrop. Comparison between contemporaneous limestones and dolomites demonstrate a general increase in Fe_T within the dolomites, predominantly present as Fe_{carb} and consistent with late stage alteration due to a through-flowing Fe-rich dolomitizing fluid. The effect of late stage dolomitization is very heterogeneous within a single bed, resulting in some samples with Fe_T enrichments and others with depletions. Either way, the Fe_{HR}/Fe_T ratio will be affected and thus we avoided sampling late stage dolomites.

All analysed carbonate samples presented here are from limestones and early dolomites. Early stage dolomitization does not cause an increase in Fe_T due to the lack of Fe-rich dolomitizing fluid in shallow burial environment[12]. Early dolomites tend to show very low Fe_T values of <0.5 wt% suggesting they record the low original Fe_T values typical of oxic carbonates[12]. Some dolomitized marls in the Saiq Plateau anoxic interval

(Supplementary Fig. 2) show similarly high Fe_T values and Fe_{HR}/Fe_T ratios to surrounding shales, suggesting that these represent primary signatures.

Supplementary References

1 Richoz, S. Stratigraphie et variations isotopiques du carbone dans le Permien supérieur et le Trias inférieur de quelques localitiés de la Néothéthys (Turquie, Oman et Iran). *Memoires de Geologie (Lausanne)* **46**, 275, (2006).

2 Krystyn, L., Richoz, S., Baud, A. & Twitchett, R. J. A unique Permian-Triassic boundary section from the Neotethyan Hawasina Basin, Central Oman Mountains. *Palaeogeography Palaeoclimatology Palaeoecology* **191**, 329-344, (2003).

3 Baud, A., Richoz, S., Beauchamp, B., Cordey, F., Grasby, S., Henderson, C. M., Krystyn, L. & Nicora, A. The Buday'ah Formation, Sultanate of Oman: A Middle Permian to Early Triassic oceanic record of the Neotethys and the late Induan microsphere bloom. *Journal of Asian Earth Sciences* **43**, 130-144, (2012).

4 Koehrer, B., Zeller, M., Aigner, T., Poeppelreiter, M., Milroy, P., Forke, H. & Al-Kindi, S. Facies and stratigraphic framework of a Khuff outcrop equivalent: Saiq and Mahil formations, Al Jabal al-Akhdar, Sultanate of Oman. *GeoArabia* **15**, 91-156, (2010).

5 Algeo, T. J. & Twitchett, R. J. Anomalous Early Triassic sediment fluxes due to elevated weathering rates and their biological consequences. *Geology* **38**, 1023-1026, (2010).

6 Twitchett, R. J., Krystyn, L., Baud, A., Wheeley, J. R. & Richoz, S. Rapid marine recovery after the end-Permian mass-extinction event in the absence of marine anoxia. *Geology* **32**, 805-808, (2004).

7 Richoz, S., Baud, A., Beauchamp, B., Grasby, S., Henderson, C. & Krystyn, L. Khuff Margin: slope to oceanic deposits (Permian-Triassic Allochthons and Exotics, Oman) in *Permo-Triassic Sequence of the Arabian Plate* (ed M. Pöpperlreiter) (EAGE Special Volume, 2014).

8 Woods, A. D. & Baud, A. Anachronistic facies from a drowned Lower Triassic carbonate platform: Lower member of the Alwa Formation (Ba'id Exotic), Oman Mountains. *Sedimentary Geology* **209**, 1-14, (2008).

9 Raiswell, R. & Canfield, D. E. Sources of iron for pyrite formation in marine sediments. *American Journal of Science* **298**, 219-245, (1998).

10 Poulton, S. W., Fralick, P. W. & Canfield, D. E. The transition to a sulphidic ocean similar to 1.84 billion years ago. *Nature* **431**, 173-177, (2004).

11 Poulton, S. W. & Canfield, D. E. Ferruginous Conditions: A Dominant Feature of the Ocean through Earth's History. *Elements* **7**, 107-112, (2011).

12 Clarkson, M. O., Poulton, S. W., Guilbaud, R. & Wood, R. Assessing the utility of Fe/Al and Fe-speciation to record water column redox conditions in carbonate-rich sediments. *Chemical Geology* **382**, 111-122, (2014).
